# A machine learning-based diagnostic model associated with knee osteoarthritis severity

**DOI:** 10.1038/s41598-020-72941-4

**Published:** 2020-09-25

**Authors:** Soon Bin Kwon, Yunseo Ku, Hyuk-Soo Han, Myung Chul Lee, Hee Chan Kim, Du Hyun Ro

**Affiliations:** 1 Interdisciplinary Program in Bioengineering, Seoul National University, Seoul, Korea; 2 Department of Biomedical Engineering, College of Medicine, Chungnam National University, Daejeon, Korea; 3 Department of Orthopedic Surgery, Seoul National University Hospital, Seoul National University College of Medicine, 101 Daehak-ro, Jongno-gu, Seoul, 110-744 Korea; 4 Institute of Medical & Biological Engineering, Medical Research Center, Seoul National University College of Medicine, Seoul, Korea; 5 Department of Biomedical Engineering, Seoul National University College of Medicine, Seoul, Korea

**Keywords:** Biomedical engineering, Diagnostic markers, Clinical trials

## Abstract

Knee osteoarthritis (KOA) is characterized by pain and decreased gait function. We aimed to find KOA-related gait features based on patient reported outcome measures (PROMs) and develop regression models using machine learning algorithms to estimate KOA severity. The study included 375 volunteers with variable KOA grades. The severity of KOA was determined using the Western Ontario and McMaster Universities Osteoarthritis Index (WOMAC). WOMAC scores were used to classify disease severity into three groups. A total of 1087 features were extracted from the gait data. An ANOVA and student’s t-test were performed and only features that were significant were selected for inclusion in the machine learning algorithm. Three WOMAC subscales (physical function, pain and stiffness) were further divided into three classes. An ANOVA was performed to determine which selected features were significantly related to the subscales. Both linear regression models and a random forest regression was used to estimate patient the WOMAC scores. Forty-three features were selected based on ANOVA and student’s t-test results. The following number of features were selected from each joint: 12 from hip, 1 feature from pelvic, 17 features from knee, 9 features from ankle, 1 feature from foot, and 3 features from spatiotemporal parameters. A significance level of < 0.0001 and < 0.00003 was set for the ANOVA and t-test, respectively. The physical function, pain, and stiffness subscales were related to 41, 10, and 16 features, respectively. Linear regression models showed a correlation of 0.723 and the machine learning algorithm showed a correlation of 0.741. The severity of KOA was predicted by gait analysis features, which were incorporated to develop an objective estimation model for KOA severity. The identified features may serve as a tool to guide rehabilitation and progress assessments. In addition, the estimation model presented here suggests an approach for clinical application of gait analysis data for KOA evaluation.

## Introduction

Knee osteoarthritis (KOA) is a leading cause of disability among older adults, affecting more than 250 million patients worldwide^1^. The economic burden associated with osteoarthritis (OA) is high with 1–2% of the gross national product spent on OA-related healthcare^2,3^. Approximately 30% of individuals aged over 60 years suffer from KOA^4^. As the proportion of older people increases in the population, it is expected that the number of patients who require KOA surgery will also rise^5^. Typical KOA symptoms include stiffness and decreased joint range of motion, which can greatly impair functional independence^6^. KOA is also characterized by pain and gait dysfunction that steadily deteriorates with disease progression^7^.

KOA therapy is aimed at reducing pain and improving gait function. The results from pain and gait disorder assessments are used to develop treatment plans, determine the effectiveness of treatment, and inform disease prognosis. Conventionally, the pain and gait disorder assessments are dependent on patient-reported outcome measures (PROMs). Even though PROMs are cost and time efficient, they are prone to bias^8,9^. Since its publication in 1988, the Western Ontario and McMaster University Osteoarthritis Index (WOMAC) has served as the gold standard for determining OA severity^10^. The WOMAC consists of three subscales: pain, stiffness and physical function with 5, 2 and 17 questions, respectively. The WOMAC index has been widely used in clinical studies^11–13^; however, it is not accessible to individuals with cognitive impairment, depression or those unwilling to provide accurate answers^8^. In addition, discordance between WOMAC scores and actual physical gait improvement has been noted^14^.

Clinical gait analysis is a powerful technique that provides objective and reliable biomechanical information, including temporal waveforms for each of the lower body joints^15^. The measurement devices for gait quantification includes 3D motion capture, force plates, instrumented mats, wearable sensors with inertial measurement unites and accelerometer^16^. Since gait dysfunction can be evaluated objectively using this method, it has been suggested as an alternative tool for measuring patient disabilities^8,14,17^. Previously^18^, we identified an association between gait analysis features and KOA radiological grade and showed successful estimation of the Kellgren-Lawerence (KL) grade using a machine learning algorithm based on key gait features.

Information is limited regarding the relationship between PROMs and kinetic and kinematic gait features. These analyses can provide potentially objective measures of symptoms and provide insight regarding the relationship between symptomatic diagnosis of disease and gait quality. Current barriers to clinical application of gait analysis include the absence of a standard method for gait evaluation and the large volume and high complexity of gait analysis data^19^. Feature extraction is widely used method to analyze complex signal. Previous studies have extracted features from gait signal and analyzed the relationship between features and KOA severity^7,20,21^. However, features reported from the most previous studies were limited to traditional features and joints. In this study, we have extracted as many feature from gait data including both traditional and engineering methods from multiple joints. Also, we anticipate that WOMAC estimation model based on gait feature would explain the biomechanical difference between the severity of KOA and provide further understanding for the relationship between KOA and gait function.

Our cross-sectional study analyzed the relationship between gait data and the WOMAC scores of KOA patients. The WOMAC indices of KOA patients without cognitive impairment, depression and who were willing to answer accurately, were included to avoid longitudinal bias and other possible inaccuracies. We hypothesized that the WOMAC index and its three subscales would closely relate to KOA patients’ gait function and that specific features would change with disease progression. Overall, our study aimed to identify the key features associated with the WOMAC index and its three subscales, and to apply these key features to develop estimation models for WOMAC to improve rehabilitation and suggest standardize application for gait analysis.

## Methods

### Participants

This study was approved by our Institutional Review Board of Seoul National University Hospital (IRB no. 1810-004-974) and were performed in accordance with relevant guidelines and regulations. Written informed consent was obtained from all participants. This study was performed using our gait lab database. The database consists of gait reports of KOA patients with various degrees of knee pain and healthy volunteers without any knee pain from 2013 to 2017. We excluded subjects based on the following criteria: (1) missing some data for both legs; (2) aged < 20 years; (3) spine disease, hip, or ankle arthritis on x-ray; (4) inflammatory or traumatic arthritis of the knee; (5) any prior bone surgery in the lower extremities; and (6) cognitive impairment or depression. A total of 375 subjects were included in our study.

### Data collection

All gait analysis data, including kinetic, kinematic and spatial–temporal data, were collected at the Human Motion Analysis Laboratory of Seoul National University Hospital following OrthoTrack 6.6 Reference Manual ^22^ with daily quality check to maintain the error within 1 mm. All data collection process was performed by an operator with 20 years of experience. The subjects has a few minutes to warm up to acclimate to the setting before placing reflective markers based on the Helen Hayes arrangement. After placing the markers, an operator asked the subjects to walk along a 9 m track. Motion data were collected using twelve charge-coupled device cameras with a three-dimensional optical motion capture system (Motion Analysis Corp., Santa Rosa, CA, USA) at a sampling frequency of 120 Hz. Two floor-embedded force plates were used to obtain the kinetic data. An average of five or six trials of the 9 m walk of the kinetic and kinematic data for each joint were used in this study.

All participants performed self-administered Korean version of WOMAC^23^ with three subscales; pain (5 questions), stiffness (2 questions) and physical function (17 questions). Each questions were answered in numeric scale ranging from 0 (no symptoms) to 4 (extreme symptom).

### Feature extraction and statistical analysis

All data analyses and classification were performed using MATLAB 2018b (MathWorks, Massachusetts). The gait features were extracted from the gait parameters, which are temporal signal of kinetic and kinematic data of hip, pelvic area, knee and ankle. These features included, but were not limited to, area under the curve, maximum value of swing phase, and minimum value of the curve. An additional 16 gait characteristics (i.e., velocity and cadence) were also selected as classification model features. Only the right leg was included to avoid statistical dependency from multiple observations of single individuals^24^. Detail information of extracted features were included in Supplementary Table S1.

To statically analyze the relationship between the WOMAC score and gait features, the severity of WOMAC was classified into three classless: mild, moderate, and severe. Each WOMAC questions are answered into 5 different answers: none (0), mild (1), moderate (2), severe (3) and extreme (4). To divide the WOMAC score into three different severities, 1.5, the midpoint between mild to moderate, and 2.5, the midpoint between moderate to severe, were chosen as the cut point and were multiplied by 24, which is the number of WOMAC questionnaires. Accordingly, WOMAC scores below 36 was classified as mild, scores between 36 and 60 were classified as moderate, and the scores above 60 were classified as severe. The WOMAC subscales were divided into three classes using the same procedure. A one-way analysis of variance (ANOVA) with a significance level of 0.0001 was performed. A student t-test was used to analyze class differences between each severity groups for features with significant difference as the result of ANOVA. For a multiple-comparison correction, a new alpha value of < 0.00003 was used as significance level according to Bonferroni correction^25^. Features that were significant for all three comparisons between each classes were selected as key features. Student’s t-test was performed again for selected key features between each severity group divided in accordance to each subscale of WOMAC.

A multiple linear regression was performed to estimate the WOMAC index and to examine its relationship with WOMAC key features and observe feasibility of the estimation model. To resolve dataset imbalances, we down-sampled the sample size to 231. A random forest algorithm^26^, an ensemble learning method constructed with multiple decision trees, was used to build the regression model for WOMAC index estimation. ‘Statistic and Machine Learning Toolbox’ from Matlab was used for the machine learning analysis.

The hold-out method was used for model validation only for random forest model. Seventy percent of the data were randomly selected to train the model and the other thirty percent of data were used for validation. The model was analyzed by observing the root mean square error (RMSE) and correlation between actual and estimated WOMAC score.

### Clinical implication

Gait function of KOA patients decreases due to typical symptoms of KOA patients such as tenderness, loss of flexibility, and swelling. This study statistically analyzed the relationship between gait data and symptomatic severity of KOA and applied machine learning algorithm for WOMAC estimation. The implication of this study were followings:

1. Provides further understanding between KOA symptoms and gait data
2. Estimation model can be applied to patients who cannot properly perform WOMAC evaluation due to cognitive impairment or other clinical problem
3. If gait analysis can be performed with more accessible technology, such as wearable sensor and pose-estimation using camera, this study can serve as foundation research for patient independent diagnosis.

## Results

Table 1 summarizes the participants’ demographic characteristics and symptomatic severity. A total of 1083 features (of 23 gait parameters) were extracted from the gait analysis dataset and 42 features (12 hip, 1 pelvic, 17 knee, 8 ankle, 1 foot, and 3 spatiotemporal) were selected according to ANOVA and t-test results. The gait parameter features included hip rotation moment, hip flexion angle, hip adduction angle, hip power, pelvic obliquity angle, knee extension moment, knee flexion angle, knee power, knee varus angle, ankle plantarflexion moment, ankle power, foot progression angle, total speed**,** duration of single limb support phase (% of gait cycle), timing of initial double limb support (% of gait cycle), and timing of weight acceptance (% of gait cycle). Physical function was significantly related to all features, with the exception of hip power. Pain differed significantly in relation to hip adduction angle, hip power, knee power, knee varus angle, ankle plantarflexion moment, and ankle power. Stiffness was significantly different in relation to hip rotation moment, hip adduction angle, knee flexion angle, and knee varus angle.

**Table 1:** Subject characteristics.

Feature	Mild (n = 140)	Moderate (n = 182)	Severe (n = 53)	p-value
Age	62.6 (9.1)	63.7 (10.2)	63.3 (10.2)	0.101
WOMAC	18.9 (11.9)	48.5 (6.8)	71.7 (10.3)	< 0.001
Physical Function	13.8 (8.9)	35.4 (5.6)	52.8 (7.8)	< 0.001
Pain	3.4 (3.2)	9.1 (2.3)	13.2 (3.7)	< 0.001
Stiffness	1.7 (1.5)	4 (1.7)	5.7 (1.7)	< 0.001

**Figure 1:**
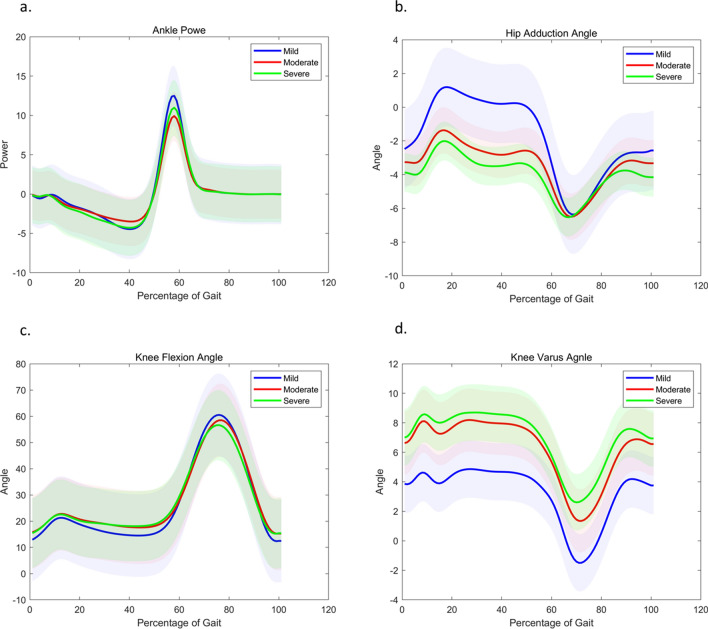
Mean values of representative gait parameters for each symptomatic severity of KOA where features were extracted from the (**a**) ankle power, (**b**) hip adduction angle, (**c**) knee flexion angle, and (**d**) knee varus angle. The shaded area represents standard deviation.

**Figure 2:**
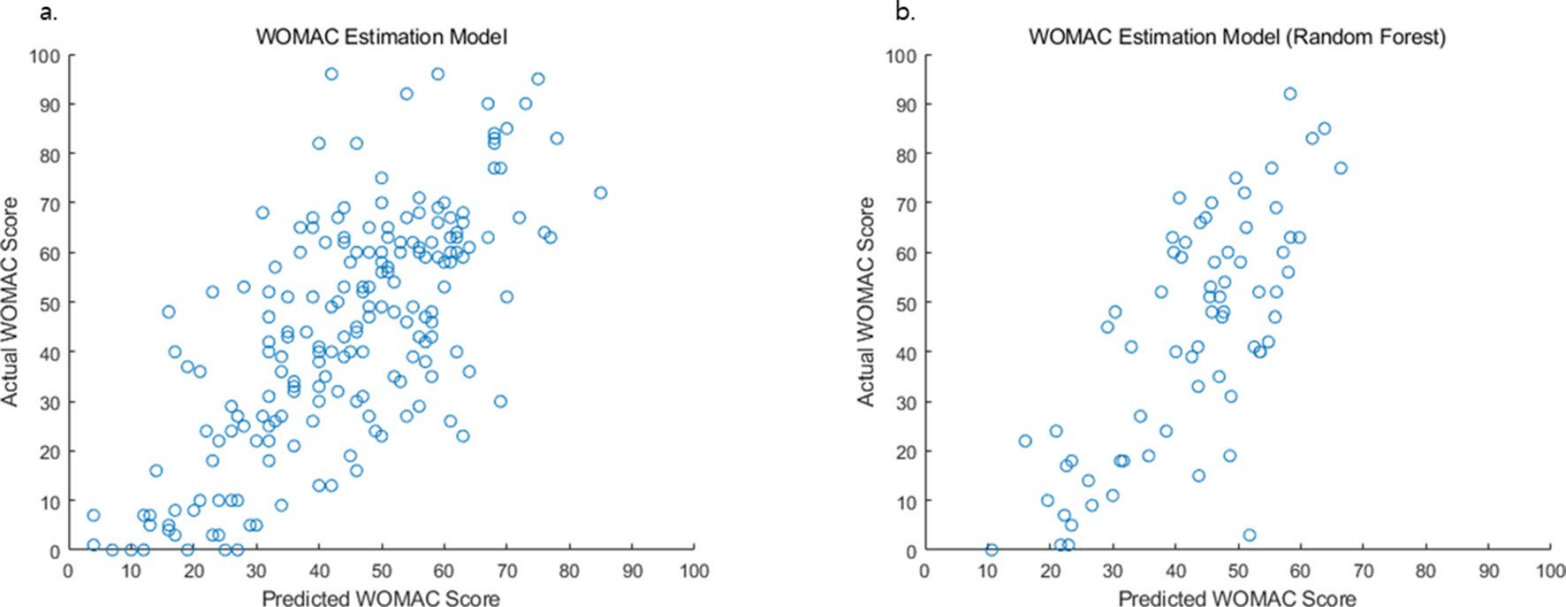
Regression result for WOMAC results using (**a**) linear regression (**b**) the random forest algorithm and identified key features.

The representative mean values of parameters for each group were divided according to WOMAC score (Fig. 1). Table 2 summarizes the key WOMAC features with mean and standard deviation. All features listed in Table 2 showed significant difference among all severity groups according to student’s t-test. Area under the curve during stance phase of hip adduction angle, variance of knee flexion angle, area under the curve of stance phase and mid-reference level of knee varus angle, and peak-to-RMS of ankle power showed most significant difference among the three groups. The RMSE for linear regression was 16.10, and RMSE for random forest regression was 17.38. The correlation between actual and estimated WOMAC score was 0.722 and 0.741, respectively for linear regression and random forest regression (Fig. 2).

**Table 2:** Mean and standard deviation of selected features significantly different for WOMAC severity groups.

Joint	Parameter	Feature	Mild	Moderate	Severe
Hip	Rotation moment	Standard deviation	2.67 (0.63)	2.39 (0.52)	2.36 (0.58)
	Flexion angle	Lower bound of Autocorrelation	− 0.44 (0.0013)	− 0.43 (0.0019)	− 0.43 (0.0022)
		Bandwidth frequency bounds	0.0025 (0.00016)	0.0024 (0.00022)	0.024 (0.00021)
	Adduction angle	**Area under the curve during stance phase**	− **1.4 (4.07)**	− **4.11 (4.99)**	− **5.23 (5.21)**
		Standard deviation of absolute value	298.18 (262.45)	425.53 (321.97)	501.01 (315.01)
	Power	Minimum value during mid-stance	− 1.36 (2.06)	− 0.91 (1.89)	0.02 (2.01)
		Maximum value during terminal stance	4.72 (3.86)	3.13 (3.51)	3.37 (2.84)
		Area under the curve	121.33 (107.22)	104.2 (106.16)	179.68 (115.38)
		Maximum − minimum	11.56 (4.92)	9.32 (4.97)	10.68 (4.16)
		Distance between stance and swing phase using dynamic time wrapping	125.88 (64.02)	96.33 (62.12)	88.94 (49.67)
		Maximum value during mid-swing	6.74 (3.16)	5.19 (3.28)	5.99 (2.7)
		Minimum value during terminal swing	− 0.37 (0.5)	− 0.45 (0.55)	− 0.78 (0.81)
Pelvic	Obliquity angle	Minimum value during terminal stance to pre-swing	3.65 (0.83)	3.19 (0.9)	3.32 (0.79)
Knee	Extension moment	Kurtosis	2.23 (0.52)	1.97 (0.45)	2.11 (0.48)
		Peak2RMS	2.14 (0.3)	1.98 (0.28)	2.02 (0.28)
	Flexion angle	**Variance**	**279.1 (109.53)**	**227.72 (110.94)**	**216.22 (116.47)**
		Standard deviation	16.37 (3.27)	14.53 (4.06)	13.93 (4.72)
		Maximum − minimum	51.4 (8.82)	46.29 (11.48)	44.3 (13.34)
		Area under the curve of power spectral density	276.99 (108.54)	225.94 (110)	214.6 (115.55)
	Power	Maximum value during terminal swing	− 7.47 (3.65)	− 5.8 (3.36)	− 6.25 (3.58)
	Varus Angle	Maximum value during mid-stance	5.36 (4.55)	8.81 (6.06)	9.34 (6.35)
		Maximum Value during Terminal Stance	5.09 (4.4)	8.46 (6.01)	8.95 (6.53)
		**Area under the curve of stance phase**	**274.47 (277.05)**	**490.8 (377.33)**	**517.3 (401.42)**
		Area under the curve	334.36 (420.53)	641.28 (530.54)	710.69 (595.35)
		Root mean square (RMS)	5.18 (3.03)	7.77 (3.9)	8.64 (4.28)
		Peak2RMS	1.73 (0.49)	1.42 (0.34)	1.45 (0.39)
		**Mid-reference level**	**308.36 (239.71)**	**534.6 (295.59)**	**573.58 (319.35)**
		Area under the curve of power spectrum	0.94 (1.26)	2.19 (2.31)	2.65 (2.38)
		Maximum Value during Terminal swing	5.39 (4.29)	7.88 (5.03)	8.5 (5.47)
		Minimum value during loading response	3.12 (4.14)	6.18 (5.52)	6.4 (5.71)
Ankle	Plantarflexion moment	Minimum value during loading response	− 0.61 (0.56)	− 0.34 (0.44)	− 0.31 (0.48)
		Maximum value during initial Swing	− 0.3 (0.11)	− 0.23 (0.14)	− 0.26 (0.11)
		Maximum − minimum	11.38 (3.68)	9.76 (3.36)	10.28 (2.81)
	Power	Kurtosis	6.73 (1.14)	6.01 (1.48)	6.12 (1.27)
		**Peak2RMS**	**3.44 (0.32)**	**3.24 (0.43)**	**3.28 (0.34)**
		Maximum − minimum	20.98 (9.02)	16.62 (8.73)	19.15 (6.99)
		Lower bound of autocorrelation	− 0.43 (0.0031)	− 0.43 (0.0030)	− 0.43 (0.0032)
		Occupied bandwidth	0.89 (0.25)	1.09 (0.35)	0.98 (0.27)
Foot	Progression angle	Average of absolute value	− 0.44 (0.0013)	− 0.44 (0.0020)	− 0.44 (0.0018)
Spatiotemporal		Total speed	85.41 (18.25)	75.43 (21.82)	81.87 (17.44)
		Duration of single limb support phase	35.5 (2.72)	33.58 (4.38)	35.34 (3.14)
		Timing of initial double limb support	14.61 (3.05)	16.37 (4.38)	14.42 (2.96)

The bolded rows are four features that showed most significant difference among each groups.

## Discussion

While previous studies^14,27,28^ have reported the relationship between spatiotemporal gait features, such as speed and stride length, and WOMAC indices of KOA or hip OA patients, this is the first study to analyze the relationship between kinetic and kinematic gait parameters and the WOMAC indices. Biomechanical intervention is recognized an alternative method to control pain and improve physical function^29^. Gait analysis provides meaningful KOA biomechanical information, but its complexity has limited its clinical applicability^19,30^. Here, we statistically analyzed key gait cycle features and identified critical KOA biomechanical information. In addition, we built linear and machine learning estimation models for the WOMAC index based on the identified features. While PROM methods are cheap, easy and quick, they are not applicable to patients who are unable or unwilling to perform the task. Despite the ability of gait analysis to provide valuable information about KOA biomechanical properties, a standardized method is not available for clinical use. Our estimation model provides objective and reliable symptomatic results and suggests utility as a consistent method for evaluating gait analysis data. Finally, we have extracted key features based on both conventional methods, such as mean value of the curve, and novel engineering methods, such as occupied bandwidth of the curve in frequency domain. Conventional features, such as peak and minimum gait data values, are limited to load or motion at a single time point during the gait cycle and do not contain information over the gait cycle^31^. We have developed methods that include information over the entire gait cycle, such as area under the curve, root mean square (RMS) and power spectrum. We also conducted detailed feature analysis during gait cycle sub-phases: loading response, mid-stance, terminal-stance and pre-swing of stance phase, and initial swing mid-swing and terminal swing of swing phase.

We identified well-known joint parameters that are specific to KOA patients and function in gait performance (listed in Table 2). Ankle dorsiflexion moment, for example, is an ankle joint movement involved in supination and pronation and three-dimensional ankle joint motions^32^. Previous studies have shown that knee varus angle changes are closely related to KOA^33,34^. Lo and colleagues reported an association between knee varus angle and knee pain during weight bearing activities, most likely due to narrowing of the medial joint space, opening of the lateral space or increased lateral soft tissue pretension. We found that hip, knee and ankle joint power, the product of torque and angular velocity, differed significantly according to WOMAC severity. Similarly, Segal et al.^35^ reported joint power differences between symptomatic KOA patients and high-functioning controls. In one of our previous studies, we have reported that difference between maximum and minimum value of both hip flexion angle and hip adduction angle were smaller in KOA patients compared with control group^36^. Weidow et al. have reported that^37^ the maximum value of hip rotation moment significantly differed between symptomatic and asymptomatic group. KOA patients was reported to have significantly lower knee flexion range of motion in swing and stance phase during gait cycle, which is in agreement with our findings^38^. McCarthy et al.^39^ claimed that knee extension moment is also an important gait characteristic to analyze the relationship between KOA and gait data. Bechard et al.^40^ reported that toe-out angle of foot progression angle was significantly smaller in patients with KOA and pelvic obliquity angle was reported to be correlated with symptoms of KOA.

In our study^18^, physical function was influenced by the greatest number of features (42 from 13 parameters), indicating that WOMAC is a comprehensive score that incorporates the movement of many joints. This is reasonable given that KOA also effects the kinetic and kinematics of hip and ankle joints. Thus, to improve the physical function of patients, it is important to train not just the knee joint but also other KOA-affected joints^41^. The results of our study provide guidelines for KOA exercise and rehabilitation (Table 2). Pain and stiffness were most related to knee-specific parameters. This pattern is demonstrated by tibiofemoral OA, which is a fairly common form of OA related to varus alignment. Tibiofemoral OA patients report higher pain levels than patellofemoral OA patients. Knee extension moment was not significantly related to pain. However, the WOMAC pain questionnaire only included one stair-related question, which may have influenced this result. In addition, the questionnaire also lacked questions related to knee adduction moment. Stiffness showed a significant relationship with knee flexion angle, a sagittal plane parameter. This is notable because the main movement of the knee, extension and flexion, is included in the sagittal plane.

A limitation of our study was that it was validated internally; to validate the model for overfitting it should be subjected to external validation. In addition, the features identified in this study were not applied to actual rehabilitation. Future studies should apply the key features to patient rehabilitation and determine the therapeutic effects.

In conclusion, we have built estimation models for the WOMAC index and have identified features associated with the WOMAC and its subscales. The features have been extracted using a feature engineering technique and statistically selected and validated. The estimation models were generated by traditional linear regression and random forest regression models. Our estimation model and list of key features represents an objective and alternative option for KOA symptom diagnosis and rehabilitation.

## Supplementary information

Supplementary Table 1.

Supplementary Table 2.

Supplementary Table 3.

Supplementary Table 4.

## Data Availability

The codes of this study for statistic process and estimation model are available at https://github.com/SBEKwon/SCI_Report
