# Author Correction: A machine learning-based diagnostic model associated with knee osteoarthritis severity

**DOI:** 10.1038/s41598-022-07058-x

**Published:** 2022-02-15

**Authors:** Soon Bin Kwon, Yunseo Ku, Hyuk-Soo Han, Myung Chul Lee, Hee Chan Kim, Du Hyun Ro

**Affiliations:** 1 Interdisciplinary Program in Bioengineering, Seoul National University, Seoul, Korea; 2 Department of Biomedical Engineering, College of Medicine, Chungnam National University, Daejeon, Korea; 3 Department of Orthopedic Surgery, Seoul National University Hospital, Seoul National University College of Medicine, 101 Daehak-ro, Jongno-gu, Seoul, 110-744 Korea; 4 Institute of Medical & Biological Engineering, Medical Research Center, Seoul National University College of Medicine, Seoul, Korea; 5 Department of Biomedical Engineering, Seoul National University College of Medicine, Seoul, Korea

Correction to: *Scientific Reports* 10.1038/s41598-020-72941-4, published online 25 September 2020

The original version of this Article contained an error in the spelling of the author Hyuk-Soo Han, which was incorrectly given as Hy uk-soo Han.

The original Article has been corrected.

