# Author Correction: A machine learning-based diagnostic model associated with knee osteoarthritis severity

**DOI:** 10.1038/s41598-024-73460-2

**Published:** 2024-09-30

**Authors:** Soon Bin Kwon, Yunseo Ku, Hyuk‑Soo Han, Myung Chul Lee, Hee Chan Kim, Du Hyun Ro

**Affiliations:** 1 Interdisciplinary Program in Bioengineering, Seoul National University, Seoul, Korea; 2 Department of Biomedical Engineering, College of Medicine, Chungnam National University, Daejeon, Korea; 3 Department of Orthopedic Surgery, Seoul National University Hospital, Seoul National University College of Medicine, 101 Daehak‑ro, Jongno‑gu, Seoul, 110‑744 Korea; 4 Institute of Medical & Biological Engineering, Medical Research Center, Seoul National University College of Medicine, Seoul, Korea; 5 Department of Biomedical Engineering, Seoul National University College of Medicine, Seoul, Korea

Correction to: *Scientific Reports*10.1038/s41598-020-72941-4, published online 25 September 2020

This Article contains an error in the Methods section, under the subheading ‘Participants’.

"Written informed consent was obtained from all participants."

should read:

“This study is a retrospective study, and informed consent was waived by the institutional review board."

