# Supplementary material for: A machine learning-based diagnostic model associated with knee osteoarthritis severity
Source: Sci Rep. 2020 Sep 25;10:15743. doi: 10.1038/s41598-020-72941-4 (PMC7519044; doi:10.1038/s41598-020-72941-4)
Supplement: Supplementary file 1 — Supplementary Table 1. [file 41598_2020_72941_MOESM1_ESM.docx]

A machine learning-based diagnostic model associated with knee osteoarthritis severity

Soon Bin Kwon,^1^ Yunseo Ku,^2^ Hyuk-soo Han^3^, Myung Chul Lee^3^, Hee Chan Kim,^1,4,5^ and Du Hyun Ro^3^

^1^Interdisciplinary Program in Bioengineering, Seoul National University, Seoul, Korea;

^2^Department of Biomedical Engineering, College of Medicine, Chungnam National University, Daejeon, Korea

^3^Department of Orthopedic Surgery, Seoul National University Hospital, Seoul National University College of Medicine;

^4^Institute of Medical & Biological Engineering, Medical Research Center, Seoul National University College of Medicine, Seoul, Korea;

^5^Department of Biomedical Engineering, Seoul National University College of Medicine, Seoul, Korea

**Supplementary Table 1.** Mean and standard deviation of extracted gait features

| Gait Parameter | Feature | Mild | Moderate | Severe |
| --- | --- | --- | --- | --- |
| Hip Adduction Moment | Maximum Value during Loading Response to Mid-Stance | 6.95(2.14) | 6.18(2.22) | 6.26(2.35) |
|  | Minimum Value During Mid-Stance | 5.72(1.88) | 5.06(1.95) | 5.15(2.35) |
|  | Maximum Value during Terminal Stance to Pre-Swing | 6.79(2.1) | 6.2(1.99) | 6.38(2.34) |
|  | Minimum Value during Pre-Swing to Initial Swing | -0.65(0.44) | -0.5(0.5) | -0.59(0.5) |
|  | Area Under the Curve of Stance Phase | 294.72(95.59) | 256.2(94.52) | 256.97(106.43) |
|  | Area Under the Curve | 293.83(93.32) | 256.44(92.85) | 256.76(104.47) |
|  | Area Under the Curve of Absolute Value During Stance Phase | 299.26(94.29) | 262.35(92.55) | 264.41(104.29) |
|  | Area Under the Curve of Absolute Value | 312.58(96.6) | 273.61(95.42) | 277.12(106.7) |
|  | Root Mean Square | 4.18(1.28) | 3.7(1.26) | 3.77(1.43) |
|  | Mean | 2.91(0.92) | 2.54(0.92) | 2.54(1.04) |
|  | Variance | 9.84(5.89) | 8.06(4.46) | 8.77(5.88) |
|  | Standard Deviation | 3(0.91) | 2.7(0.89) | 2.78(1.03) |
|  | Kurtosis | 1.32(0.14) | 1.4(0.22) | 1.44(0.22) |
|  | Peak2RMS | 1.72(0.09) | 1.77(0.12) | 1.78(0.14) |
|  | Maximum - Minimum | 7.98(2.34) | 7.21(2.29) | 7.44(2.53) |
|  | Upper Bound of Autocorrelation | 0.44(0) | 0.44(0) | 0.44(0) |
|  | Lower Bound of Autocorrelation | -0.44(0) | -0.44(0) | -0.44(0) |
|  | Absolute Value of Area Under the Curve During Stance Phase | 294.72(95.59) | 256.69(93.19) | 256.98(106.41) |
|  | Absolute Value of Area Under the Curve | 293.83(93.32) | 256.83(91.78) | 257.37(102.92) |
|  | Root Mean Square of Absolute Value | 4.18(1.28) | 3.7(1.26) | 3.77(1.43) |
|  | Mean of Absolute Value | 3.1(0.96) | 2.71(0.95) | 2.75(1.06) |
|  | Variance of Absolute Value | 8.64(5.32) | 7.09(3.92) | 7.62(5.13) |
|  | Standard Deviation of Absolute Value | 2.81(0.87) | 2.53(0.84) | 2.59(0.97) |
|  | Kurtosis of Absolute Value | 1.31(0.14) | 1.39(0.2) | 1.45(0.25) |
|  | Peak2RMS of Absolute Value | 1.72(0.09) | 1.77(0.12) | 1.78(0.14) |
|  | Absolute Maximum - Absolute Minimum | 7.16(2.15) | 6.47(2.11) | 6.61(2.33) |
|  | Time instants of the mid-reference level crossings | 1.56(0.54) | 1.67(0.55) | 1.69(0.5) |
|  | Mid-reference level | 3.24(1) | 3.01(1) | 3.1(1.19) |
|  | Minimum distance between signals | 281.48(96.61) | 242.7(90.79) | 243.66(105.12) |
|  | upper cumulative sums drifted beyond five standard deviations above a target mean | 24.25(12.17) | 24.02(9.59) | 24.62(7.19) |
|  | lower cumulative sums drifted beyond five standard deviations below a target mean | 38.56(27.93) | 43.88(28.67) | 40.27(28.39) |
|  | index at which the mean of curve changes most significantly | 55.32(8.19) | 54.14(10.98) | 53.47(9.13) |
|  | Area Under the Curve of Power Spectral Density | 9.76(5.84) | 7.99(4.42) | 8.69(5.83) |
|  | Maximum Value of Area Under the Curve of Power Spectral Density | 880.7(531.1) | 719.78(408.39) | 773.48(538.8) |
|  | Occupied bandwidth | 2.21(0.3) | 2.08(0.34) | 2.08(0.33) |
|  | Lower bandwidth frequency bounds | 0(0) | 0(0) | 0(0) |
|  | Upper bandwidth frequency bounds | 2.21(0.3) | 2.08(0.34) | 2.09(0.33) |
|  | Power stored in bandwidth | 16228.19(25569.71) | 10435.49(12138.13) | 13558.85(17955.61) |
|  | Power spectrum | 0.7(0.51) | 0.56(0.37) | 0.57(0.45) |
|  | Max Spectrum Frequencies | 37.2(27.58) | 29.68(21.62) | 29.23(24.01) |
|  | Area Under the Curve of Power Measurment in dB | -Inf | -Inf | -Inf |
|  | Area Under the Curve of Absoulute Value of Power Measurment in dB | Inf(NaN) | Inf(NaN) | Inf(NaN) |
|  | Maximum Value of Power Measurment in dB | -Inf | -Inf | -Inf |
|  | Number of Peaks | 2.54(0.75) | 2.38(0.83) | 2.29(0.66) |
|  | Number of Peaks of absoulte Value | 3.93(1.05) | 3.8(1.19) | 3.73(1.03) |
| Hip Rotation Moment | Maximum Value of the Curve | 1.13(0.59) | 1.01(0.62) | 1.07(0.55) |
|  | Minimum Value of the Curve | -0.53(0.35) | -0.51(0.56) | -0.48(0.26) |
|  | Maximum Value during Pre-Swing to Initial Swing | 0.11(0.36) | 0.16(0.38) | 0.22(0.51) |
|  | Area Under the Curve of Stance Phase | 14.34(21.05) | 13.83(30.12) | 18.74(22.37) |
|  | Area Under the Curve | 12.72(20.76) | 12.56(30.11) | 17.35(22.28) |
|  | Area Under the Curve of Absolute Value During Stance Phase | 32.85(13.59) | 32.88(22.95) | 33.43(17.93) |
|  | Area Under the Curve of Absolute Value | 37.56(14.88) | 37.98(24.44) | 39.25(19.96) |
|  | Root Mean Square | 0.52(0.22) | 0.51(0.34) | 0.51(0.26) |
|  | Mean | 0.13(0.21) | 0.13(0.3) | 0.17(0.22) |
|  | Variance | 0.26(0.22) | 0.27(0.53) | 0.26(0.24) |
|  | Standard Deviation | 0.48(0.19) | 0.45(0.26) | 0.46(0.2) |
|  | Kurtosis | 2.67(0.63) | 2.39(0.52) | 2.36(0.58) |
|  | Peak2RMS | 2.31(0.28) | 2.21(0.25) | 2.2(0.29) |
|  | Maximum - Minimum | 1.66(0.67) | 1.52(0.8) | 1.55(0.62) |
|  | Upper Bound of Autocorrelation | 0.44(0) | 0.44(0) | 0.44(0) |
|  | Lower Bound of Autocorrelation | -0.44(0) | -0.44(0) | -0.44(0) |
|  | Absolute Value of Area Under the Curve During Stance Phase | 19.84(15.93) | 22.59(24.21) | 21.3(19.9) |
|  | Absolute Value of Area Under the Curve | 18.9(15.31) | 21.93(24.12) | 20.18(19.7) |
|  | Root Mean Square of Absolute Value | 0.52(0.22) | 0.51(0.34) | 0.51(0.26) |
|  | Mean of Absolute Value | 0.37(0.15) | 0.38(0.24) | 0.39(0.2) |
|  | Variance of Absolute Value | 0.16(0.16) | 0.17(0.49) | 0.14(0.19) |
|  | Standard Deviation of Absolute Value | 0.36(0.17) | 0.34(0.24) | 0.33(0.17) |
|  | Kurtosis of Absolute Value | 2.91(0.82) | 2.68(0.68) | 2.75(0.85) |
|  | Peak2RMS of Absolute Value | 2.31(0.28) | 2.21(0.25) | 2.2(0.29) |
|  | Absolute Maximum - Absolute Minimum | 1.21(0.54) | 1.13(0.7) | 1.11(0.5) |
|  | Time instants of the mid-reference level crossings | 1.62(0.66) | 1.65(0.78) | 1.53(0.74) |
|  | Mid-reference level | 0.32(0.44) | 0.26(0.53) | 0.32(0.38) |
|  | Minimum distance between signals | 33.63(14.47) | 33.67(21.65) | 34.75(17.81) |
|  | upper cumulative sums drifted beyond five standard deviations above a target mean | 8.09(17.41) | 10.9(17.5) | 10.53(18.13) |
|  | lower cumulative sums drifted beyond five standard deviations below a target mean | 22.05(15.2) | 21.82(17.26) | 23.2(15.86) |
|  | index at which the mean of curve changes most significantly | 34.89(10.61) | 37.12(13.62) | 37.33(10.93) |
|  | Area Under the Curve of Power Spectral Density | 0.26(0.22) | 0.27(0.52) | 0.25(0.24) |
|  | Maximum Value of Area Under the Curve of Power Spectral Density | 18.85(15.26) | 21.79(47.17) | 20.66(21.83) |
|  | Occupied bandwidth | 1.97(0.55) | 1.96(0.51) | 2.02(0.44) |
|  | Lower bandwidth frequency bounds | 0(0) | 0(0) | 0(0) |
|  | Upper bandwidth frequency bounds | 1.98(0.55) | 1.97(0.51) | 2.02(0.44) |
|  | Power stored in bandwidth | 11.05(23.3) | 42.48(442.82) | 14.38(37.92) |
|  | Power spectrum | 0.01(0.01) | 0.01(0.04) | 0.01(0.01) |
|  | Max Spectrum Frequencies | 0.3(0.45) | 0.48(2.48) | 0.3(0.62) |
|  | Area Under the Curve of Power Measurment in dB | -Inf | -Inf | -Inf |
|  | Area Under the Curve of Absoulute Value of Power Measurment in dB | Inf(NaN) | Inf(NaN) | Inf(NaN) |
|  | Maximum Value of Power Measurment in dB | -Inf | -Inf | -Inf |
|  | Number of Peaks | 3.34(1.09) | 3.17(1.26) | 3.02(0.99) |
|  | Number of Peaks of absoulte Value | 4.65(1.33) | 4.39(1.54) | 4.25(1.24) |
| Hip Extension Moment | Maximum Value during Loading Response to Mid-Stance | 4.27(1.82) | 3.8(1.81) | 4.66(2.04) |
|  | Minimum Value of the Curve | -4.24(1.84) | -3.66(1.9) | -3.6(1.44) |
|  | Maximum Value during Mid-Swing to Terminal Swing | 2.17(0.97) | 1.81(0.95) | 2.14(0.99) |
|  | Area Under the Curve of Stance Phase | -10(95.93) | 3.52(95.09) | 55.4(94.77) |
|  | Area Under the Curve | -12.07(91.3) | -1.42(90.87) | 47.15(90.94) |
|  | Area Under the Curve of Absolute Value During Stance Phase | 169.97(60.6) | 156.05(63.78) | 176.37(64.93) |
|  | Area Under the Curve of Absolute Value | 216.4(73.44) | 196.4(76.73) | 226.72(80.57) |
|  | Root Mean Square | 2.61(0.91) | 2.36(0.94) | 2.7(0.95) |
|  | Mean | -0.1(0.9) | 0(0.9) | 0.49(0.9) |
|  | Variance | 6.9(4.53) | 5.72(4.66) | 7.21(4.48) |
|  | Standard Deviation | 2.49(0.84) | 2.23(0.87) | 2.54(0.87) |
|  | Kurtosis | 2.06(0.23) | 2(0.3) | 1.93(0.23) |
|  | Peak2RMS | 1.96(0.17) | 1.93(0.19) | 1.9(0.18) |
|  | Maximum - Minimum | 8.58(3.03) | 7.48(3.03) | 8.39(2.78) |
|  | Upper Bound of Autocorrelation | 0.43(0) | 0.44(0) | 0.44(0) |
|  | Lower Bound of Autocorrelation | -0.43(0) | -0.44(0) | -0.44(0) |
|  | Absolute Value of Area Under the Curve During Stance Phase | 73.39(62.3) | 73.25(60.52) | 87.7(65.37) |
|  | Absolute Value of Area Under the Curve | 70.18(59.37) | 69.99(57.75) | 81.03(62.03) |
|  | Root Mean Square of Absolute Value | 2.61(0.91) | 2.36(0.94) | 2.7(0.95) |
|  | Mean of Absolute Value | 2.17(0.73) | 1.96(0.77) | 2.27(0.81) |
|  | Variance of Absolute Value | 2.45(1.93) | 2.04(1.79) | 2.43(1.67) |
|  | Standard Deviation of Absolute Value | 1.46(0.56) | 1.31(0.57) | 1.47(0.52) |
|  | Kurtosis of Absolute Value | 2.27(0.39) | 2.24(0.43) | 2.17(0.37) |
|  | Peak2RMS of Absolute Value | 1.96(0.17) | 1.93(0.19) | 1.9(0.18) |
|  | Absolute Maximum - Absolute Minimum | 5.11(1.9) | 4.55(1.91) | 5.1(1.84) |
|  | Time instants of the mid-reference level crossings | 1.63(0.57) | 1.5(0.66) | 1.47(0.57) |
|  | Mid-reference level | -0.53(1.65) | -0.24(1.54) | 0.19(1.41) |
|  | Minimum distance between signals | 202.59(70.55) | 182.78(76.14) | 207.09(79.14) |
|  | upper cumulative sums drifted beyond five standard deviations above a target mean | 9.51(25.15) | 7.74(21.37) | 8.98(19.66) |
|  | lower cumulative sums drifted beyond five standard deviations below a target mean | 24.61(5.47) | 25.18(8.74) | 25.29(6.89) |
|  | index at which the mean of curve changes most significantly | 30.85(16.15) | 32.61(16.36) | 35.53(13.79) |
|  | Area Under the Curve of Power Spectral Density | 6.88(4.52) | 5.7(4.65) | 7.17(4.45) |
|  | Maximum Value of Area Under the Curve of Power Spectral Density | 630.34(407.14) | 527.35(438.18) | 664.64(426.41) |
|  | Occupied bandwidth | 1.81(0.4) | 1.91(0.42) | 1.94(0.49) |
|  | Lower bandwidth frequency bounds | 0(0) | 0(0) | 0(0) |
|  | Upper bandwidth frequency bounds | 1.81(0.4) | 1.91(0.42) | 1.94(0.49) |
|  | Power stored in bandwidth | 8174.18(12365.13) | 6711.74(22175.2) | 9242.96(11451.57) |
|  | Power spectrum | 0.34(0.33) | 0.26(0.3) | 0.22(0.21) |
|  | Max Spectrum Frequencies | 17.61(17.48) | 13.97(17.28) | 10.58(12.22) |
|  | Area Under the Curve of Power Measurment in dB | -Inf | -Inf | -Inf |
|  | Area Under the Curve of Absoulute Value of Power Measurment in dB | Inf(NaN) | Inf(NaN) | Inf(NaN) |
|  | Maximum Value of Power Measurment in dB | -Inf | -Inf | -Inf |
|  | Number of Peaks | 1.99(0.78) | 2.23(0.87) | 2.15(0.65) |
|  | Number of Peaks of absoulte Value | 2.99(0.86) | 3.2(0.99) | 3.18(0.75) |
| Hip Flexion Angle | Maximum Value during Loading Response to Mid-Stance | 37.18(6.65) | 37.92(8.21) | 41.31(7.8) |
|  | Minimum Value of the Curve | -1.57(7.42) | 0.37(9.63) | 3.06(10.35) |
|  | Maximum Value during Mid-Swing to Terminal Swing | 39.29(6.62) | 39.76(8.03) | 43.05(7.63) |
|  | Area Under the Curve of Stance Phase | 1035.74(458.93) | 1145.01(576.08) | 1302.52(615.55) |
|  | Area Under the Curve | 2100.28(643.79) | 2189.05(804.45) | 2470.72(868.22) |
|  | Area Under the Curve of Absolute Value During Stance Phase | 1142.76(339.13) | 1245.01(463.4) | 1350.78(561.74) |
|  | Area Under the Curve of Absolute Value | 2209.61(519.57) | 2294.58(682.6) | 2527.57(792.42) |
|  | Root Mean Square | 25.59(5.22) | 26.17(6.67) | 28.64(7.42) |
|  | Mean | 21.16(6.44) | 22.05(8.04) | 24.87(8.67) |
|  | Variance | 194.79(56.18) | 180.35(73.51) | 184.06(57.53) |
|  | Standard Deviation | 13.81(2.06) | 13.14(2.77) | 13.36(2.37) |
|  | Kurtosis | 1.65(0.1) | 1.7(0.14) | 1.69(0.13) |
|  | Peak2RMS | 1.55(0.1) | 1.55(0.13) | 1.54(0.15) |
|  | Maximum - Minimum | 40.94(5.85) | 39.54(7.66) | 40.07(6.89) |
|  | Upper Bound of Autocorrelation | 0.44(0) | 0.44(0) | 0.43(0) |
|  | Lower Bound of Autocorrelation | -0.44(0.0013) | -0.43(0.0019) | -0.43(0.0022) |
|  | Absolute Value of Area Under the Curve During Stance Phase | 1040.47(448.02) | 1154.63(556.45) | 1302.52(615.55) |
|  | Absolute Value of Area Under the Curve | 2100.28(643.79) | 2189.05(804.45) | 2470.72(868.22) |
|  | Root Mean Square of Absolute Value | 25.59(5.22) | 26.17(6.67) | 28.64(7.42) |
|  | Mean of Absolute Value | 22.24(5.21) | 23.09(6.83) | 25.43(7.92) |
|  | Variance of Absolute Value | 161.7(51.42) | 150.74(63.13) | 167.82(54.32) |
|  | Standard Deviation of Absolute Value | 12.55(2.07) | 12(2.6) | 12.73(2.43) |
|  | Kurtosis of Absolute Value | 1.59(0.13) | 1.66(0.17) | 1.67(0.17) |
|  | Peak2RMS of Absolute Value | 1.55(0.1) | 1.55(0.13) | 1.54(0.15) |
|  | Absolute Maximum - Absolute Minimum | 37.04(5.75) | 35.92(6.98) | 37.93(6.67) |
|  | Time instants of the mid-reference level crossings | 1.57(0.5) | 1.51(0.5) | 1.56(0.6) |
|  | Mid-reference level | 18.31(6.48) | 19.81(8.01) | 22.99(8.36) |
|  | Minimum distance between signals | 1243.19(191.39) | 1172.84(261.6) | 1194.73(218.08) |
|  | upper cumulative sums drifted beyond five standard deviations above a target mean | 84.12(20.48) | 81.95(24.89) | 88.53(13.15) |
|  | lower cumulative sums drifted beyond five standard deviations below a target mean | 24.98(0.24) | 25.11(1.68) | 25.02(0.13) |
|  | index at which the mean of curve changes most significantly | 75.54(7.4) | 75.89(10.01) | 72.95(15.45) |
|  | Area Under the Curve of Power Spectral Density | 195.02(56.24) | 180.55(73.64) | 184.27(57.6) |
|  | Maximum Value of Area Under the Curve of Power Spectral Density | 18077.87(5374.18) | 16576.57(7114.01) | 16994.38(5344.69) |
|  | Occupied bandwidth | 1.59(0.12) | 1.61(0.13) | 1.6(0.13) |
|  | Lower bandwidth frequency bounds | 0.0025(0.00016) | 0.0024(0.00022) | 0.024(0.00021) |
|  | Upper bandwidth frequency bounds | 1.59(0.12) | 1.61(0.13) | 1.6(0.13) |
|  | Power stored in bandwidth | 5291868.24(3056354.7) | 4803217.78(4560213.07) | 4657315.83(2715334.88) |
|  | Power spectrum | 2.51(2.65) | 4.14(5.74) | 5.33(11.96) |
|  | Max Spectrum Frequencies | 186.01(218.67) | 310.92(459.52) | 422.63(920.7) |
|  | Area Under the Curve of Power Measurment in dB | -223446.83(16316.28) | -221985.67(16454.54) | -218774.9(23036.65) |
|  | Area Under the Curve of Absoulute Value of Power Measurment in dB | 233196.48(16013.79) | 232339.35(16073.72) | 229033.08(23259.74) |
|  | Maximum Value of Power Measurment in dB | 20.33(4.57) | 21.53(5.5) | 22.23(5.78) |
|  | Number of Peaks | 1.21(0.44) | 1.15(0.43) | 1.22(0.46) |
|  | Number of Peaks of absoulte Value | 1.75(0.6) | 1.64(0.64) | 1.64(0.62) |
| Hip Abduction Moment | Maximum Value during Loading Response to Mid-Stance | 0.02(2.04) | -0.53(2.36) | -0.37(2.59) |
|  | Minimum Value during Terminal Stance | -2.33(2.37) | -2.59(2.59) | -2.64(2.47) |
|  | Maximum Value during Pre-Swing to Initial Swing | 0.6(2.28) | 0.8(2.73) | 0.94(2.98) |
|  | Minimum Value during Terminal Swing | 1.93(4.35) | -0.45(4.67) | -0.82(5.38) |
|  | Area Under the Curve of Stance Phase | -1.4(4.07) | -4.11(4.99) | -5.23(5.21) |
|  | Area Under the Curve | 0.92(3.95) | -1.03(4.18) | -1.16(4.22) |
|  | Area Under the Curve of Absolute Value During Stance Phase | -3.9(3.84) | -4.49(4.12) | -5.13(4.66) |
|  | Area Under the Curve of Absolute Value | -28.89(251.42) | -183.75(303.3) | -221.37(326.48) |
|  | Root Mean Square | -179.12(359.39) | -341.97(403.62) | -397.3(430.81) |
|  | Mean | 228.74(141.32) | 293.24(211.83) | 331.68(222.02) |
|  | Variance | 401.24(209.65) | 476.11(279.39) | 537.63(272.39) |
|  | Standard Deviation | 4.62(2.11) | 5.29(2.78) | 5.98(2.71) |
|  | Kurtosis | -1.8(3.59) | -3.42(4.03) | -3.97(4.31) |
|  | Peak2RMS | 9.83(7.26) | 7.87(6.97) | 9.03(6.88) |
|  | Maximum - Minimum | 2.93(1.11) | 2.58(1.11) | 2.81(1.09) |
|  | Upper Bound of Autocorrelation | 2.15(0.57) | 2.22(0.55) | 2.02(0.48) |
|  | Lower Bound of Autocorrelation | 1.86(0.32) | 1.78(0.36) | 1.67(0.29) |
|  | Absolute Value of Area Under the Curve During Stance Phase | 9.73(3.48) | 8.56(3.37) | 9.02(3.01) |
|  | Absolute Value of Area Under the Curve | 0.44(0.01) | 0.44(0.01) | 0.44(0) |
|  | Mean of Absolute Value | -0.44(0.01) | -0.44(0.01) | -0.44(0) |
|  | Variance of Absolute Value | 193.56(162.29) | 269.32(230.27) | 312.28(239.1) |
|  | Standard Deviation of Absolute Value | 298.18(262.45) | 425.53(321.97) | 501.01(315.01) |
|  | Kurtosis of Absolute Value | 4.01(2.1) | 4.76(2.79) | 5.37(2.72) |
|  | Peak2RMS of Absolute Value | 5.39(4.03) | 5.33(4.53) | 6.84(5.71) |
|  | Absolute Maximum - Absolute Minimum | 2.17(0.83) | 2.13(0.9) | 2.43(0.98) |
|  | Time instants of the mid-reference level crossings | 2.42(0.68) | 2.37(0.69) | 2.03(0.48) |
|  | Mid-reference level | 1.86(0.32) | 1.78(0.36) | 1.67(0.29) |
|  | Minimum distance between signals | 7.53(2.55) | 7.27(2.87) | 7.91(2.7) |
|  | upper cumulative sums drifted beyond five standard deviations above a target mean | 1.59(0.72) | 1.8(1.04) | 1.56(0.71) |
|  | lower cumulative sums drifted beyond five standard deviations below a target mean | -2.68(4.05) | -4.35(4.1) | -4.34(4.32) |
|  | index at which the mean of curve changes most significantly | 235.01(119.98) | 203.18(118.79) | 230.69(121.48) |
|  | Area Under the Curve of Power Spectral Density | 18.95(18.53) | 29.38(25) | 26.91(25.93) |
|  | Maximum Value of Area Under the Curve of Power Spectral Density | 28.13(24.62) | 22.89(17.61) | 22.98(18.07) |
|  | Occupied bandwidth | 56.97(12.45) | 55.57(16.01) | 55.38(13.08) |
|  | Lower bandwidth frequency bounds | 9.75(7.2) | 7.81(6.92) | 8.96(6.82) |
|  | Upper bandwidth frequency bounds | 806.45(655.11) | 650.59(638.55) | 776.88(661.68) |
|  | Power stored in bandwidth | 1.94(0.46) | 1.93(0.45) | 2(0.52) |
|  | Power spectrum | 0(0) | 0(0) | 0(0) |
|  | Max Spectrum Frequencies | 1.94(0.46) | 1.93(0.45) | 2.01(0.52) |
|  | Area Under the Curve of Power Measurment in dB | 17258.05(27247.67) | 13015.61(30465.91) | 16321.03(27474.22) |
|  | Area Under the Curve of Absoulute Value of Power Measurment in dB | 0.54(0.63) | 0.89(1.17) | 1.03(1.09) |
|  | Maximum Value of Power Measurment in dB | 34.6(50.16) | 64.3(87.42) | 73.56(82.86) |
|  | Number of Peaks | -241067.88(18820.04) | -240067.58(20304.21) | -239737.57(25568.1) |
|  | Number of Peaks of absoulte Value | 245691.6(18401.14) | 245126.72(19980.28) | 245469.06(25250.98) |
|  | Maximum Value during Mis-Stance | 11.99(5.69) | 14.09(6.77) | 15.49(5.96) |
|  | Maximum Value during Terminal Stance to Pre-Swing | 2.63(0.93) | 2.84(1.08) | 2.84(1.37) |
|  | Minimum Value during Pre-Swing to Initial Swing | 3.41(1.01) | 3.42(1.23) | 3.36(1.32) |
|  | Maximum Value during Terminal Swing | 1.69(4.5) | -0.9(5) | -1.59(5.92) |
| Hip Power | Maximum Value during Mid-Stance | 4.65(3.16) | 4.23(2.93) | 6(3.5) |
|  | Minimum Value during Mid-Stance | -1.42(2.06) | -0.87(1.89) | -0.07(2.01) |
|  | Maximum Value during Terminal Stance | 4.72(3.86) | 3.13(3.51) | 3.37(2.84) |
|  | Minimum Value during Pre-Swing to Initial Swing | -3.82(2.2) | -3.34(2.36) | -3.06(2.14) |
|  | Area Under the Curve of Stance Phase | 53.44(90.85) | 53.31(88.07) | 106.99(95.3) |
|  | Area Under the Curve | 121.33(107.22) | 104.2(106.16) | 179.68(115.38) |
|  | Area Under the Curve of Absolute Value During Stance Phase | 175.94(74.96) | 152.82(79.13) | 183.03(76.08) |
|  | Area Under the Curve of Absolute Value | 248(99.67) | 213.81(104.85) | 263.16(106.64) |
|  | Root Mean Square | 3.23(1.3) | 2.75(1.35) | 3.35(1.34) |
|  | Mean | 1.21(1.07) | 1.09(1.05) | 1.77(1.14) |
|  | Variance | 9.6(7.12) | 7.14(7.47) | 8.7(6.56) |
|  | Standard Deviation | 2.88(1.15) | 2.39(1.2) | 2.75(1.08) |
|  | Kurtosis | 2.75(0.59) | 2.64(0.6) | 2.56(0.44) |
|  | Peak2RMS | 2.35(0.28) | 2.27(0.3) | 2.23(0.25) |
|  | Maximum - Minimum | 11.56(4.92) | 9.32(4.97) | 10.68(4.16) |
|  | Upper Bound of Autocorrelation | 0.43(0) | 0.43(0) | 0.43(0) |
|  | Lower Bound of Autocorrelation | -0.43(0) | -0.43(0) | -0.43(0) |
|  | Absolute Value of Area Under the Curve During Stance Phase | 81.79(66.3) | 79.97(64.69) | 117.13(82.26) |
|  | Absolute Value of Area Under the Curve | 129.4(97.41) | 119.9(93.81) | 178.99(112.78) |
|  | Root Mean Square of Absolute Value | 3.23(1.3) | 2.75(1.35) | 3.35(1.34) |
|  | Mean of Absolute Value | 2.47(0.99) | 2.13(1.04) | 2.62(1.06) |
|  | Variance of Absolute Value | 5.09(4.11) | 3.79(4.28) | 5.12(3.86) |
|  | Standard Deviation of Absolute Value | 2.08(0.87) | 1.74(0.88) | 2.1(0.85) |
|  | Kurtosis of Absolute Value | 2.84(0.92) | 2.63(0.89) | 2.54(0.69) |
|  | Peak2RMS of Absolute Value | 2.35(0.28) | 2.27(0.3) | 2.23(0.25) |
|  | Absolute Maximum - Absolute Minimum | 7.59(3.3) | 6.21(3.35) | 7.42(3.08) |
|  | Time instants of the mid-reference level crossings | 3.1(1.2) | 3.02(1.17) | 3.13(1) |
|  | Mid-reference level | 1.23(1.91) | 0.89(1.84) | 1.52(1.94) |
|  | Distance between Stance and Swing Phase using Dynamic Time Wrapping | 125.88(64.02) | 96.33(62.12) | 88.94(49.67) |
|  | upper cumulative sums drifted beyond five standard deviations above a target mean | 27.85(22.88) | 28.84(24.46) | 21.35(21.78) |
|  | lower cumulative sums drifted beyond five standard deviations below a target mean | 25.46(9.57) | 26.43(10.78) | 25.55(10.17) |
|  | index at which the mean of curve changes most significantly | 45.62(19.59) | 41.44(21.82) | 37.44(20.25) |
|  | Area Under the Curve of Power Spectral Density | 9.53(7.07) | 7.09(7.42) | 8.63(6.51) |
|  | Maximum Value of Area Under the Curve of Power Spectral Density | 562.54(406.49) | 441.01(458.56) | 551.57(427.81) |
|  | Occupied bandwidth | 1.18(0.25) | 1.26(0.37) | 1.44(0.48) |
|  | Lower bandwidth frequency bounds | 0(0) | 0(0) | 0(0) |
|  | Upper bandwidth frequency bounds | 1.19(0.25) | 1.26(0.37) | 1.44(0.48) |
|  | Power stored in bandwidth | 9368.86(13056.99) | 7618.31(29671.31) | 8834.17(13957.98) |
|  | Power spectrum | 0.43(0.43) | 0.32(0.46) | 0.28(0.29) |
|  | Max Spectrum Frequencies | 13.21(12.37) | 9.85(12.94) | 9.66(10.25) |
|  | Area Under the Curve of Power Measurment in dB | -Inf | -Inf | -Inf |
|  | Area Under the Curve of Absoulute Value of Power Measurment in dB | Inf(NaN) | Inf(NaN) | Inf(NaN) |
|  | Maximum Value of Power Measurment in dB | -Inf | -Inf | -Inf |
|  | Number of Peaks | 3.2(0.9) | 3.52(1.21) | 3.15(0.91) |
|  | Number of Peaks of absoulte Value | 5.22(1.15) | 5.5(1.53) | 5.18(1.17) |
|  | Maximum Value during Loading Response | 4.81(3.09) | 4.42(2.85) | 6.15(3.34) |
|  | Minimum Value during Terminal Stance to Pre-Swing | -4.18(2.34) | -3.47(2.43) | -3.21(2.26) |
|  | Maximum Value during Mid-Swing | 6.74(3.16) | 5.19(3.28) | 5.99(2.7) |
|  | Minimum Value during Terminal Swing | -0.37(0.5) | -0.45(0.55) | -0.78(0.81) |
| Hip Rotation Angle | Maximum Value during Mis-Stance | -2.77(7.73) | -1.54(7.14) | -1.48(7.15) |
|  | Minimum Value during Mid-Stance to Terminal Stance | -6.9(8.09) | -5.83(7.1) | -5.15(7.44) |
|  | Maximum Value during Terminal Stance to Pre-Swing | -3.8(7.98) | -3.21(7.18) | -2.48(7.82) |
|  | Minimum Value during Pre-Swing to Initial Swing | -12.07(7.71) | -10.37(6.83) | -8.85(7.02) |
|  | Area Under the Curve of Stance Phase | -368.95(490.38) | -303.39(446.21) | -247.08(462.33) |
|  | Area Under the Curve | -614.63(725.83) | -478.12(645.59) | -414.4(660.21) |
|  | Area Under the Curve of Absolute Value During Stance Phase | 507.76(352.19) | 448.87(313.56) | 410.46(329.79) |
|  | Area Under the Curve of Absolute Value | 819.42(507.69) | 706.51(431.35) | 645.24(460.38) |
|  | Root Mean Square | 8.94(5.02) | 7.76(4.21) | 7.05(4.58) |
|  | Mean | -6.14(7.26) | -4.77(6.46) | -4.14(6.6) |
|  | Variance | 14.86(10.53) | 13.73(9.65) | 10.58(7.27) |
|  | Standard Deviation | 3.64(1.26) | 3.5(1.22) | 3.07(1.08) |
|  | Kurtosis | 2.54(0.67) | 2.67(0.85) | 2.54(0.81) |
|  | Peak2RMS | 1.8(0.39) | 1.84(0.4) | 1.75(0.34) |
|  | Maximum - Minimum | 13.46(4.23) | 13.08(4.35) | 11.13(3.43) |
|  | Upper Bound of Autocorrelation | 0.43(0.01) | 0.43(0.01) | 0.43(0.01) |
|  | Lower Bound of Autocorrelation | -0.43(0.01) | -0.43(0.01) | -0.43(0.01) |
|  | Absolute Value of Area Under the Curve During Stance Phase | 487.47(371.88) | 422.25(335.28) | 392.51(344.97) |
|  | Absolute Value of Area Under the Curve | 769.41(557.83) | 638.89(486.1) | 593.01(502.62) |
|  | Root Mean Square of Absolute Value | 8.94(5.02) | 7.76(4.21) | 7.05(4.58) |
|  | Mean of Absolute Value | 8.2(5.08) | 7.06(4.31) | 6.44(4.6) |
|  | Variance of Absolute Value | 12.17(10.16) | 9.57(6.91) | 8.08(6.73) |
|  | Standard Deviation of Absolute Value | 3.23(1.31) | 2.92(1.04) | 2.62(1.11) |
|  | Kurtosis of Absolute Value | 2.54(0.75) | 2.67(0.87) | 2.5(0.84) |
|  | Peak2RMS of Absolute Value | 1.8(0.39) | 1.84(0.4) | 1.75(0.34) |
|  | Absolute Maximum - Absolute Minimum | 11.79(4.26) | 10.77(3.46) | 9.44(3.57) |
|  | Time instants of the mid-reference level crossings | 2.66(1.49) | 2.63(1.38) | 2.58(1.4) |
|  | Mid-reference level | -7.23(7.34) | -5.5(6.64) | -4.85(6.54) |
|  | Minimum distance between signals | 214.46(138.8) | 220.39(120.77) | 187.66(112.15) |
|  | upper cumulative sums drifted beyond five standard deviations above a target mean | 22.32(24.17) | 22.36(27.31) | 23.87(27.94) |
|  | lower cumulative sums drifted beyond five standard deviations below a target mean | 40.95(24.64) | 38.23(21.09) | 34.24(21.9) |
|  | index at which the mean of curve changes most significantly | 58.25(22.94) | 59.31(23.91) | 60.27(19.59) |
|  | Area Under the Curve of Power Spectral Density | 14.77(10.47) | 13.65(9.59) | 10.51(7.24) |
|  | Maximum Value of Area Under the Curve of Power Spectral Density | 991.87(859.83) | 899.65(726.11) | 679.06(548.63) |
|  | Occupied bandwidth | 1.73(0.47) | 1.72(0.5) | 1.81(0.58) |
|  | Lower bandwidth frequency bounds | 0(0) | 0(0) | 0(0) |
|  | Upper bandwidth frequency bounds | 1.73(0.47) | 1.73(0.5) | 1.81(0.58) |
|  | Power stored in bandwidth | 29041.41(52316.57) | 23290.2(37960.95) | 13194.51(17611.46) |
|  | Power spectrum | 2.71(3.77) | 2.14(2.71) | 2.08(3.3) |
|  | Max Spectrum Frequencies | 202.49(281.73) | 156.6(208.02) | 152.01(250.5) |
|  | Area Under the Curve of Power Measurment in dB | -213831.64(20382.47) | -213552.2(17470.96) | -213770.15(29220.6) |
|  | Area Under the Curve of Absoulute Value of Power Measurment in dB | 221021.88(20120.15) | 220476.27(17542.12) | 220355.76(29835.48) |
|  | Maximum Value of Power Measurment in dB | 18.54(7.55) | 17.62(7.23) | 16.83(6.97) |
|  | Number of Peaks | 3.95(1.03) | 4.15(1.09) | 4.36(1.08) |
|  | Number of Peaks of absoulte Value | 4.5(1.32) | 4.85(1.41) | 5.11(1.52) |
|  | Maximum Value during Loading Response to Mid-Stance | -1.94(7.77) | -0.07(7.2) | -0.09(6.94) |
|  | Minimum Value during Initial Swing to Mid-Swing | -12.43(7.39) | -10.87(6.67) | -9.15(6.8) |
|  | Maximum Value during Terminal Swing | -2.14(8.03) | -0.69(7.47) | -0.93(6.25) |
| Pelvic Tilt Angle | Maximum Value during Loading Response to Mid-Stance | 3.66(1.56) | 3.86(1.81) | 4.31(1.8) |
|  | Minimum Value during Terminal Stance to Pre-Swing | 5.22(1.64) | 5.41(2.01) | 5.64(1.92) |
|  | Maximum Value during Mid-Swing | 1.2(0.7) | 0.97(0.66) | 0.96(0.58) |
|  | Area Under the Curve of Stance Phase | 13.51(4.23) | 14.12(5.45) | 16.07(6.19) |
|  | Area Under the Curve | 12(4.16) | 12.54(5.35) | 14.33(6.24) |
|  | Area Under the Curve of Absolute Value During Stance Phase | 13.39(4.13) | 14.12(5.41) | 16.22(6.33) |
|  | Area Under the Curve of Absolute Value | 12.13(4.17) | 12.22(5.27) | 14.42(5.99) |
|  | Root Mean Square | 789.79(271.67) | 829.85(357.74) | 932.56(405.58) |
|  | Mean | 1229.67(410.23) | 1265.79(528.35) | 1474.33(608.17) |
|  | Variance | 789.79(271.67) | 831.71(353.45) | 943.55(378.8) |
|  | Standard Deviation | 1229.67(410.23) | 1268.11(522.82) | 1492.1(562.29) |
|  | Kurtosis | 12.34(4.09) | 12.74(5.21) | 14.98(5.59) |
|  | Peak2RMS | 12.3(4.1) | 12.66(5.28) | 14.74(6.08) |
|  | Maximum - Minimum | 0.95(1.12) | 1.34(1.88) | 1.43(1.23) |
|  | Upper Bound of Autocorrelation | 0.89(0.39) | 1.03(0.52) | 1.1(0.46) |
|  | Lower Bound of Autocorrelation | 2.24(0.39) | 2.21(0.39) | 2.16(0.34) |
|  | Absolute Value of Area Under the Curve During Stance Phase | 1.14(0.07) | 1.17(0.16) | 1.13(0.08) |
|  | Absolute Value of Area Under the Curve | 3.13(1.21) | 3.54(1.56) | 3.76(1.45) |
|  | Root Mean Square of Absolute Value | 0.43(0.01) | 0.43(0.01) | 0.43(0.01) |
|  | Mean of Absolute Value | -0.43(0.01) | -0.43(0.01) | -0.43(0.01) |
|  | Variance of Absolute Value | 789.79(271.67) | 831.1(354.81) | 943.55(378.8) |
|  | Standard Deviation of Absolute Value | 1229.67(410.23) | 1267.38(524.51) | 1492.1(562.29) |
|  | Kurtosis of Absolute Value | 12.3(4.1) | 12.68(5.23) | 14.92(5.62) |
|  | Peak2RMS of Absolute Value | 0.95(1.12) | 1.33(1.88) | 1.43(1.23) |
|  | Absolute Maximum - Absolute Minimum | 0.89(0.39) | 1.03(0.52) | 1.1(0.46) |
|  | Time instants of the mid-reference level crossings | 2.24(0.39) | 2.21(0.39) | 2.16(0.34) |
|  | Mid-reference level | 1.14(0.07) | 1.17(0.16) | 1.13(0.08) |
|  | Minimum distance between signals | 3.13(1.21) | 3.53(1.56) | 3.76(1.45) |
|  | upper cumulative sums drifted beyond five standard deviations above a target mean | 2.52(1.2) | 2.17(1.11) | 2.16(1.13) |
|  | lower cumulative sums drifted beyond five standard deviations below a target mean | 12.11(4.16) | 12.51(5.35) | 14.52(6.05) |
|  | index at which the mean of curve changes most significantly | 52.69(38.9) | 63.85(52.65) | 63.43(40.47) |
|  | Area Under the Curve of Power Spectral Density | 33.63(19.91) | 33.74(21.3) | 31.67(17) |
|  | Maximum Value of Area Under the Curve of Power Spectral Density | 30.73(28.21) | 29.59(28.34) | 36.62(28.69) |
|  | Occupied bandwidth | 55.38(21.77) | 51.44(22.34) | 54.13(20.84) |
|  | Lower bandwidth frequency bounds | 0.94(1.11) | 1.33(1.88) | 1.41(1.22) |
|  | Upper bandwidth frequency bounds | 71.99(106.63) | 104.99(175.52) | 110.26(100.35) |
|  | Power stored in bandwidth | 1.67(0.56) | 1.65(0.57) | 1.66(0.61) |
|  | Power spectrum | 0(0) | 0(0) | 0(0) |
|  | Max Spectrum Frequencies | 1.67(0.56) | 1.65(0.57) | 1.66(0.61) |
|  | Area Under the Curve of Power Measurment in dB | 251.6(1493.09) | 624.31(3167.99) | 397(753.93) |
|  | Area Under the Curve of Absoulute Value of Power Measurment in dB | 4.5(2.78) | 5.2(3.87) | 6.87(5.89) |
|  | Maximum Value of Power Measurment in dB | 341.77(212.42) | 394.11(295.85) | 523.41(450.47) |
|  | Number of Peaks | -258821.85(18052.73) | -256726.1(19080.01) | -251588.09(22031.98) |
|  | Number of Peaks of absoulte Value | 268229.14(18040.72) | 266229.88(18630.52) | 261872.63(22308.11) |
|  | Minimum Value druing Loading Response to Mid-Stance | 24.34(3.24) | 24.18(5.3) | 25.88(3.91) |
|  | Maximum Value during Terminal Stance | 3.01(1.05) | 2.84(1.03) | 2.87(1.12) |
|  | Minimum Value during Initial Swing to Mid-Swing | 3.01(1.05) | 2.86(1.07) | 2.87(1.12) |
|  | Maximum Value during Mid-Swing to Terminal Swing | 11.38(4.22) | 11.51(5.4) | 13.67(5.91) |
| Pelvic Obliquity Angle | Maximum Value during Mid-Stance | 2.21(0.41) | 2.11(0.33) | 2.15(0.36) |
|  | Minimum value during Terminal Stance to Pre-Swing | 3.65(0.83) | 3.19(0.9) | 3.32(0.79) |
|  | Maximum Value during Pre-Swing to Initial Swing | -5.34(6.98) | -2.78(7.69) | -2.02(6.69) |
|  | Minimum Value during Terminal Swing | 1.94(2.24) | 1.36(2.41) | 1.5(2.61) |
|  | Area Under the Curve of Stance Phase | -1.29(2.1) | -2.27(2.74) | -2.46(2.9) |
|  | Area Under the Curve | 0.59(2.11) | 0.61(2.6) | 1.22(2.85) |
|  | Area Under the Curve of Absolute Value During Stance Phase | -0.37(2.25) | -0.33(2.49) | -0.54(2.46) |
|  | Area Under the Curve of Absolute Value | -5.42(125.8) | -53.37(151.44) | -54.74(161.31) |
|  | Root Mean Square | -24.03(188) | -63.39(216.19) | -62.24(213.19) |
|  | Mean | 117.93(73.53) | 144.65(95.1) | 150.08(107.82) |
|  | Variance | 190.23(105.9) | 221.56(133.7) | 236.01(141.32) |
|  | Standard Deviation | 2.19(1.1) | 2.54(1.4) | 2.71(1.53) |
|  | Kurtosis | -0.24(1.88) | -0.63(2.16) | -0.62(2.13) |
|  | Peak2RMS | 2.48(2.17) | 3.37(4.77) | 4.81(5.64) |
|  | Maximum - Minimum | 1.45(0.62) | 1.6(0.91) | 1.92(1.07) |
|  | Upper Bound of Autocorrelation | 2.12(0.47) | 1.97(0.48) | 1.89(0.39) |
|  | Lower Bound of Autocorrelation | 1.84(0.27) | 1.8(0.28) | 1.75(0.25) |
|  | Absolute Value of Area Under the Curve During Stance Phase | 4.97(2.11) | 5.15(2.62) | 6.06(3.12) |
|  | Absolute Value of Area Under the Curve | 0.44(0) | 0.44(0) | 0.44(0) |
|  | Root Mean Square of Absolute Value | -0.44(0) | -0.44(0) | -0.44(0) |
|  | Mean of Absolute Value | 92.6(85) | 122.62(103.37) | 121.17(118.82) |
|  | Variance of Absolute Value | 141.52(125.56) | 171.62(145.51) | 155.54(157.33) |
|  | Standard Deviation of Absolute Value | 1.9(1.06) | 2.21(1.34) | 2.36(1.41) |
|  | Kurtosis of Absolute Value | 1.31(1.22) | 1.73(1.77) | 2.12(2.88) |
|  | Peak2RMS of Absolute Value | 1.05(0.45) | 1.18(0.58) | 1.27(0.71) |
|  | Absolute Maximum - Absolute Minimum | 2.21(0.49) | 2.11(0.57) | 2.08(0.51) |
|  | Time instants of the mid-reference level crossings | 1.84(0.27) | 1.8(0.28) | 1.75(0.25) |
|  | Mid-reference level | 3.63(1.5) | 3.91(1.77) | 4.22(2.12) |
|  | Minimum distance between signals | 2.02(1.2) | 1.78(0.96) | 1.62(0.91) |
|  | upper cumulative sums drifted beyond five standard deviations above a target mean | -0.46(1.94) | -0.86(2.29) | -0.62(2.23) |
|  | lower cumulative sums drifted beyond five standard deviations below a target mean | 115.62(58.96) | 134.65(99.12) | 164.59(106.21) |
|  | index at which the mean of curve changes most significantly | 23.29(29.4) | 33.97(32.86) | 35.27(34.2) |
|  | Area Under the Curve of Power Spectral Density | 20.3(11.91) | 21.63(10.38) | 22.11(11.08) |
|  | Maximum Value of Area Under the Curve of Power Spectral Density | 47.11(19.47) | 49.26(18.85) | 52(16.98) |
|  | Occupied bandwidth | 2.46(2.15) | 3.35(4.74) | 4.78(5.6) |
|  | Lower bandwidth frequency bounds | 210.38(200.32) | 305.88(469.57) | 449.33(558.97) |
|  | Upper bandwidth frequency bounds | 1.89(0.5) | 1.83(0.47) | 2.03(0.48) |
|  | Power stored in bandwidth | 0(0) | 0(0) | 0(0) |
|  | Power spectrum | 1.9(0.5) | 1.83(0.47) | 2.03(0.48) |
|  | Max Spectrum Frequencies | 1315.24(2980.4) | 4596.56(19058.75) | 7559.56(20767.55) |
|  | Area Under the Curve of Power Measurment in dB | 0.14(0.23) | 0.23(0.32) | 0.25(0.41) |
|  | Area Under the Curve of Absoulute Value of Power Measurment in dB | 9.44(17.34) | 15.78(22.94) | 16.52(29.43) |
|  | Maximum Value of Power Measurment in dB | -260608.76(18294.92) | -257393.57(20211.56) | -256315.62(26770.16) |
|  | Number of Peaks | 262267.25(17924.95) | 259829.44(19910.47) | 258825.88(26483.31) |
|  | Number of Peaks of absoulte Value | 5.22(6.46) | 6.93(7.82) | 6.87(7.5) |
|  | Maximum Value during Loading Response | 2.76(0.9) | 2.67(1) | 2.49(0.88) |
|  | Maximum Value during Terminal Stance | 3.64(1.03) | 3.57(1.17) | 3.33(1) |
|  | Minimum Value during Pre-Swing to Initial Swing | 0(0) | 0(0) | 0(0) |
|  | Maximum Value during Terminal Swing | 1.92(2.27) | 1.35(2.41) | 1.48(2.61) |
| Pelvic Rotation Angle | Maximum Value during Loading Response to Mid-Stance | 0.8(4.02) | -1.59(4.5) | -2.19(4.64) |
|  | Minimum Value during Terminal Stance | -7.06(4.17) | -7.36(4.27) | -7.44(3.93) |
|  | Maximum Value during Pre-Swing to Initial Swing | -2.13(3.64) | -2.62(4.06) | -3.12(4.51) |
|  | Minimum Value during Terminal Swing | 4.33(3.38) | 3.84(3.65) | 3.8(3.47) |
|  | Area Under the Curve of Stance Phase | -2.37(4.29) | -2.33(4.24) | -3.27(4.29) |
|  | Area Under the Curve | 1.3(3.72) | 1.68(4.13) | 1.3(3.7) |
|  | Area Under the Curve of Absolute Value During Stance Phase | -0.94(3.32) | -1.94(3.28) | -1.93(3.8) |
|  | Area Under the Curve of Absolute Value | 69.83(223.29) | 61.75(228.94) | 33.48(205.35) |
|  | Root Mean Square | 47.31(323.24) | 17.14(323.33) | -14.95(322.83) |
|  | Mean | 222.19(133.95) | 225.66(131.08) | 219.07(95.99) |
|  | Variance | 328.81(169.7) | 332.65(165.62) | 335.85(159.55) |
|  | Standard Deviation | 3.79(1.83) | 3.82(1.75) | 3.89(1.76) |
|  | Kurtosis | 0.49(3.23) | 0.19(3.23) | -0.13(3.23) |
|  | Peak2RMS | 7.18(6.21) | 7.28(7.12) | 8(7.15) |
|  | Maximum - Minimum | 2.48(1.01) | 2.47(1.1) | 2.59(1.15) |
|  | Upper Bound of Autocorrelation | 1.82(0.47) | 1.83(0.58) | 1.9(0.5) |
|  | Lower Bound of Autocorrelation | 1.74(0.23) | 1.74(0.23) | 1.81(0.24) |
|  | Absolute Value of Area Under the Curve During Stance Phase | 7.81(3.15) | 7.75(3.18) | 8.37(3.24) |
|  | Absolute Value of Area Under the Curve | 0.43(0.01) | 0.44(0.01) | 0.43(0.01) |
|  | Mean of Absolute Value | -0.43(0.01) | -0.44(0.01) | -0.43(0.01) |
|  | Variance of Absolute Value | 175.72(153.89) | 185.59(147.06) | 169.47(118.57) |
|  | Standard Deviation of Absolute Value | 250.64(208.57) | 255.96(197.45) | 259.26(189.71) |
|  | Kurtosis of Absolute Value | 3.29(1.69) | 3.32(1.65) | 3.36(1.59) |
|  | Peak2RMS of Absolute Value | 4.06(3.9) | 3.88(3.5) | 4.43(4.46) |
|  | Absolute Maximum - Absolute Minimum | 1.83(0.84) | 1.81(0.78) | 1.92(0.88) |
|  | Time instants of the mid-reference level crossings | 1.96(0.54) | 1.98(0.6) | 2.06(0.58) |
|  | Mid-reference level | 1.74(0.23) | 1.74(0.23) | 1.81(0.24) |
|  | Minimum distance between signals | 5.95(2.5) | 5.92(2.33) | 6.37(2.45) |
|  | upper cumulative sums drifted beyond five standard deviations above a target mean | 1.6(0.68) | 1.61(0.8) | 1.76(0.92) |
|  | lower cumulative sums drifted beyond five standard deviations below a target mean | 0.29(3.33) | -0.01(3.44) | -0.39(3.19) |
|  | index at which the mean of curve changes most significantly | 213.02(101.03) | 209.72(110.56) | 217.48(123.58) |
|  | Area Under the Curve of Power Spectral Density | 16.26(19.85) | 16.96(14.72) | 16.38(17.02) |
|  | Maximum Value of Area Under the Curve of Power Spectral Density | 34.18(15.71) | 35.89(17.35) | 36.27(16.5) |
|  | Occupied bandwidth | 43.99(15.45) | 47.72(15.26) | 44.55(16.88) |
|  | Lower bandwidth frequency bounds | 7.14(6.2) | 7.23(7.06) | 7.95(7.1) |
|  | Upper bandwidth frequency bounds | 658.82(586.95) | 659.24(687.88) | 711.98(692.44) |
|  | Power stored in bandwidth | 2.04(0.46) | 2.12(0.49) | 2.09(0.47) |
|  | Power spectrum | 0(0) | 0(0) | 0(0) |
|  | Max Spectrum Frequencies | 2.04(0.46) | 2.12(0.49) | 2.09(0.47) |
|  | Area Under the Curve of Power Measurment in dB | 10975.99(22073.89) | 13881.55(37981.48) | 14812.29(28381.06) |
|  | Area Under the Curve of Absoulute Value of Power Measurment in dB | 0.52(0.95) | 0.54(0.7) | 0.57(0.69) |
|  | Maximum Value of Power Measurment in dB | 32.65(66.19) | 33.31(48.51) | 34.15(45.35) |
|  | Number of Peaks | -250346.88(21837.88) | -244802.99(22673.58) | -243245.64(25095.87) |
|  | Number of Peaks of absoulte Value | 254415.46(21754.08) | 249263.58(22525.15) | 248027.28(24378.22) |
|  | Minimum Value during Loading Response | 10.85(6.24) | 11.48(6.15) | 11.97(5.79) |
|  | Minimum Value During Mid-Stance | 2.49(0.98) | 2.71(0.95) | 2.65(0.91) |
|  | Maximum Value Druing Mid-Stance to Terminal Stance | 3.31(1.14) | 3.52(1.17) | 3.53(1.12) |
|  | Minimum Value During Pre-Swing | 4.05(3.44) | 3.27(3.44) | 3.26(3.49) |
|  | Maximum Value during Initial Swing to Mid-Swing | 1.59(3.47) | 1.34(3.55) | 0.87(3.2) |
| Tibia Torsion Angle | Maximum Value during Loading Response to Mid-Stance | 3.25(3.54) | 3.09(3.96) | 2.66(3.52) |
|  | Minimum Value during Terminal Stance | -2.64(4.23) | -2.64(4.08) | -3.52(4.22) |
|  | Maximum Value during Pre-Swing to Initial Swing | -0.78(3.72) | -0.93(3.43) | -1.21(3.97) |
|  | Minimum Value during Terminal Swing | -8.9(10.03) | -6.48(10.28) | -7.39(7.97) |
|  | Area Under the Curve of Stance Phase | -10.4(10.17) | -7.74(10.3) | -8.34(8.25) |
|  | Area Under the Curve | -5.83(10.05) | -3(10.46) | -3.64(8.26) |
|  | Area Under the Curve of Absolute Value During Stance Phase | -17.23(10.92) | -14.11(10.88) | -14.77(7.69) |
|  | Area Under the Curve of Absolute Value | -674.31(644.01) | -518.9(680.98) | -555.09(499.34) |
|  | Root Mean Square | -1167.2(1003.07) | -884.18(1020.99) | -954.83(757.69) |
|  | Mean | 774.67(520.93) | 702.15(496.41) | 649.79(372.2) |
|  | Variance | 1297.44(835.26) | 1131.69(753.77) | 1074.28(589.79) |
|  | Standard Deviation | 13.58(8.23) | 11.92(7.41) | 11.4(5.73) |
|  | Kurtosis | -11.69(10.03) | -8.87(10.2) | -9.58(7.57) |
|  | Peak2RMS | 15.15(13.2) | 14.59(12.43) | 14.3(11.97) |
|  | Maximum - Minimum | 3.61(1.46) | 3.52(1.49) | 3.51(1.43) |
|  | Upper Bound of Autocorrelation | 2.32(0.52) | 2.29(0.61) | 2.32(0.83) |
|  | Lower Bound of Autocorrelation | 1.58(0.34) | 1.59(0.32) | 1.58(0.28) |
|  | Absolute Value of Area Under the Curve During Stance Phase | 12.95(4.93) | 12.53(4.94) | 12.44(4.69) |
|  | Absolute Value of Area Under the Curve | 0.43(0.01) | 0.43(0.01) | 0.43(0.01) |
|  | Mean of Absolute Value | -0.43(0.01) | -0.43(0.01) | -0.43(0.01) |
|  | Variance of Absolute Value | 765.34(531.81) | 681.01(517.91) | 634.91(390.63) |
|  | Standard Deviation of Absolute Value | 1271.77(865.69) | 1087.24(800.02) | 1052.02(612.73) |
|  | Kurtosis of Absolute Value | 12.99(8.36) | 11.33(7.54) | 10.76(5.9) |
|  | Peak2RMS of Absolute Value | 13.68(12.72) | 11.84(11.47) | 12.26(9.08) |
|  | Absolute Maximum - Absolute Minimum | 3.4(1.46) | 3.13(1.43) | 3.26(1.28) |
|  | Time instants of the mid-reference level crossings | 2.33(0.56) | 2.29(0.55) | 2.29(0.81) |
|  | Mid-reference level | 1.58(0.34) | 1.59(0.32) | 1.58(0.28) |
|  | Minimum distance between signals | 12.15(5.02) | 11.2(4.73) | 11.54(4.18) |
|  | upper cumulative sums drifted beyond five standard deviations above a target mean | 2.3(1.41) | 2.28(1.2) | 2.04(1.07) |
|  | lower cumulative sums drifted beyond five standard deviations below a target mean | -12.49(10.35) | -9.43(10.41) | -9.99(7.52) |
|  | index at which the mean of curve changes most significantly | 237.17(137.63) | 245.22(143.39) | 256.48(142.02) |
|  | Area Under the Curve of Power Spectral Density | 30.28(13.29) | 31.61(13.33) | 26.38(5.49) |
|  | Maximum Value of Area Under the Curve of Power Spectral Density | 32.52(33.23) | 25.09(31.28) | 30.22(36.62) |
|  | Occupied bandwidth | 63.08(18.62) | 58.28(23.05) | 60.24(23.34) |
|  | Lower bandwidth frequency bounds | 15.08(13.16) | 14.54(12.39) | 14.25(11.94) |
|  | Upper bandwidth frequency bounds | 1079.33(1085.67) | 1088.78(1022.74) | 1130.95(1036.46) |
|  | Power stored in bandwidth | 1.69(0.49) | 1.64(0.41) | 1.66(0.4) |
|  | Power spectrum | 0(0) | 0(0) | 0(0) |
|  | Max Spectrum Frequencies | 1.7(0.49) | 1.64(0.41) | 1.66(0.4) |
|  | Area Under the Curve of Power Measurment in dB | 37284.02(89644.91) | 35574.6(73074.74) | 34695.04(70513.5) |
|  | Area Under the Curve of Absoulute Value of Power Measurment in dB | 4.57(6.04) | 3.65(5.92) | 2.9(3.48) |
|  | Maximum Value of Power Measurment in dB | 361.57(475.19) | 284.3(470.02) | 224.67(272.47) |
|  | Number of Peaks | -205890.65(24581.62) | -206677.35(20740.63) | -207265.88(27622.89) |
|  | Number of Peaks of absoulte Value | 213912.93(24018.57) | 214308.04(20666.76) | 214451.19(27774.59) |
|  | Minimum Value during Mid-Stance | 21.1(7.34) | 19.87(7.43) | 19.34(7.4) |
|  | Maximum Value druing Mid-Stance | 4.38(1.18) | 4.28(1.35) | 4.35(1.38) |
|  | Maximum Value during Terminal Stance to Initial Swing | 4.83(1.28) | 4.76(1.41) | 4.73(1.42) |
|  | Minimum Value during Initial Swing to Mid-Swing | -13.82(10.03) | -11.68(10.29) | -12.49(7.6) |
|  | Maximum Value during Mid-Swing to Terminal Swing | -10.05(10.09) | -7.68(10.52) | -8.48(7.65) |
|  | Minimum Value during Terminal Swing | -5.96(10.12) | -3.09(10.4) | -3.8(8.4) |
| Knee Extension Moment | Maximum Value from Loading Response to Terminal Stance | 4.44(2.6) | 3.78(2.7) | 3.31(2.41) |
|  | Minimum Value During Terminal Stance | 0.35(2.14) | 0.8(2.12) | 0.44(2.21) |
|  | Maximum value from Pre-Swing to Initial Swing | 2.25(1.47) | 2.49(1.83) | 2.42(1.71) |
|  | Minimum value During Terminal Swing | -1.71(0.62) | -1.48(0.61) | -1.62(0.63) |
|  | Area Under the Curve of Stance Phase | 117.42(103.34) | 117.71(117.28) | 99.69(111.05) |
|  | Area Under the Curve | 98.38(102.61) | 100.91(114.93) | 83.43(108.16) |
|  | Area Under the Curve of Absolute Value During Stance Phase | 150.03(77.07) | 146.71(90.67) | 131.61(87.76) |
|  | Area Under the Curve of Absolute Value | 177.34(80.41) | 170.65(95.35) | 159.72(93.14) |
|  | Root Mean Square | 2.27(1.05) | 2.13(1.2) | 1.96(1.13) |
|  | Mean | 0.96(1.02) | 0.99(1.14) | 0.81(1.07) |
|  | Variance | 4.34(4.11) | 3.76(3.87) | 3.34(3.36) |
|  | Standard Deviation | 1.92(0.82) | 1.72(0.89) | 1.63(0.83) |
|  | Kurtosis | 2.23(0.52) | 1.97(0.45) | 2.11(0.48) |
|  | Peak2RMS | 2.14(0.3) | 1.98(0.28) | 2.02(0.28) |
|  | Maximum - Minimum | 6.58(2.78) | 5.65(2.76) | 5.45(2.51) |
|  | Upper Bound of Autocorrelation | 0.43(0) | 0.43(0) | 0.43(0.01) |
|  | Lower Bound of Autocorrelation | -0.43(0) | -0.43(0) | -0.43(0.01) |
|  | Absolute Value of Area Under the Curve During Stance Phase | 132.5(82.97) | 136.22(95.02) | 115.24(94.48) |
|  | Absolute Value of Area Under the Curve | 117.51(79.82) | 123.44(90.17) | 103.62(88.61) |
|  | Mean of Absolute Value | 1.77(0.8) | 1.7(0.95) | 1.59(0.92) |
|  | Variance of Absolute Value | 2.51(2.62) | 2.22(2.62) | 1.73(2) |
|  | Standard Deviation of Absolute Value | 1.42(0.7) | 1.29(0.75) | 1.13(0.67) |
|  | Kurtosis of Absolute Value | 2.55(0.81) | 2.2(0.72) | 2.36(0.76) |
|  | Peak2RMS of Absolute Value | 2.12(0.3) | 1.99(0.28) | 2.02(0.28) |
|  | Absolute Maximum - Absolute Minimum | 4.78(2.25) | 4.17(2.28) | 3.8(1.97) |
|  | Time instants of the mid-reference level crossings | 2.08(0.98) | 2(1.08) | 2.16(1.08) |
|  | Mid-reference level | 1.27(1.61) | 1.07(1.47) | 0.67(1.17) |
|  | Minimum distance between signals | 146.25(76.1) | 143.58(92.66) | 123.88(88.53) |
|  | upper cumulative sums drifted beyond five standard deviations above a target mean | 7.91(14.71) | 14.98(19.16) | 15.71(20.39) |
|  | lower cumulative sums drifted beyond five standard deviations below a target mean | 47.62(23.1) | 50.38(25.92) | 47.8(27.37) |
|  | index at which the mean of curve changes most significantly | 52.7(18.05) | 57.08(17.8) | 59.25(19.46) |
|  | Area Under the Curve of Power Spectral Density | 4.29(4.06) | 3.72(3.83) | 3.31(3.32) |
|  | Maximum Value of Area Under the Curve of Power Spectral Density | 311.36(287.55) | 295.28(329.67) | 261.29(293.19) |
|  | Occupied bandwidth | 2.45(0.44) | 2.32(0.5) | 2.22(0.56) |
|  | Lower bandwidth frequency bounds | 0(0) | 0(0) | 0(0) |
|  | Upper bandwidth frequency bounds | 2.45(0.44) | 2.32(0.5) | 2.22(0.56) |
|  | Power stored in bandwidth | 3433.91(9671.72) | 3189.02(8186.39) | 2490.93(5198.55) |
|  | Power spectrum | 0.13(0.21) | 0.16(0.24) | 0.15(0.21) |
|  | Max Spectrum Frequencies | 7.74(13.44) | 10.03(15.28) | 9.04(13.54) |
|  | Area Under the Curve of Power Measurment in dB | -Inf | -Inf | -Inf |
|  | Area Under the Curve of Absoulute Value of Power Measurment in dB | Inf(NaN) | Inf(NaN) | Inf(NaN) |
|  | Maximum Value of Power Measurment in dB | -Inf | -Inf | -Inf |
|  | Number of Peaks | 2.5(0.82) | 2.32(0.95) | 2.4(0.76) |
|  | Number of Peaks of absoulte Value | 3.95(1.15) | 3.56(1.24) | 3.76(1.05) |
| Knee Adduction Moment | Maximum Value Druing Mid-Stance | 4.28(1.79) | 4.69(2.17) | 4.99(2.34) |
|  | Minimum Value during Terminal Stance | 3.59(1.57) | 3.85(2.01) | 4.24(2.21) |
|  | Maximum Value during Pre-Swing | 4.18(1.77) | 4.47(2.08) | 4.83(2.37) |
|  | Minimum Value druing Pre-Swing to Initial Swing | -0.46(0.23) | -0.4(0.38) | -0.47(0.29) |
|  | Area Under the Curve of Stance Phase | 184.82(80.16) | 198.58(97.73) | 212.57(108.5) |
|  | Area Under the Curve | 186.07(78.4) | 197.97(95.33) | 211.75(106.13) |
|  | Area Under the Curve of Absolute Value During Stance Phase | 187.16(78.94) | 203.81(90.27) | 215.94(104.76) |
|  | Area Under the Curve of Absolute Value | 197.99(80.05) | 212.41(91.44) | 225.82(105.49) |
|  | Root Mean Square | 2.59(1.08) | 2.83(1.23) | 3(1.42) |
|  | Mean | 1.85(0.78) | 1.96(0.94) | 2.1(1.05) |
|  | Variance | 3.92(3.2) | 4.79(3.42) | 5.56(4.19) |
|  | Standard Deviation | 1.83(0.76) | 2.01(0.86) | 2.15(0.99) |
|  | Kurtosis | 1.39(0.2) | 1.38(0.2) | 1.4(0.21) |
|  | Peak2RMS | 1.72(0.11) | 1.74(0.13) | 1.74(0.13) |
|  | Maximum - Minimum | 4.94(1.94) | 5.3(2.15) | 5.63(2.39) |
|  | Upper Bound of Autocorrelation | 0.44(0) | 0.44(0) | 0.44(0) |
|  | Lower Bound of Autocorrelation | -0.44(0) | -0.44(0) | -0.44(0) |
|  | Absolute Value of Area Under the Curve During Stance Phase | 184.83(80.12) | 201.8(90.88) | 213.32(106.99) |
|  | Absolute Value of Area Under the Curve | 186.07(78.4) | 200.58(89.68) | 212.25(105.1) |
|  | Root Mean Square of Absolute Value | 2.59(1.08) | 2.83(1.23) | 3(1.42) |
|  | Mean of Absolute Value | 1.96(0.79) | 2.11(0.91) | 2.24(1.05) |
|  | Variance of Absolute Value | 3.44(2.91) | 4.27(3.13) | 4.96(3.82) |
|  | Standard Deviation of Absolute Value | 1.7(0.74) | 1.89(0.84) | 2.01(0.98) |
|  | Kurtosis of Absolute Value | 1.36(0.21) | 1.38(0.26) | 1.41(0.28) |
|  | Peak2RMS of Absolute Value | 1.72(0.11) | 1.74(0.13) | 1.74(0.13) |
|  | Absolute Maximum - Absolute Minimum | 4.45(1.86) | 4.84(2.05) | 5.11(2.34) |
|  | Time instants of the mid-reference level crossings | 1.27(0.49) | 1.34(0.55) | 1.36(0.62) |
|  | Mid-reference level | 1.94(0.86) | 2.16(1.03) | 2.38(1.13) |
|  | Minimum distance between signals | 174.84(80.93) | 196.62(91) | 208.24(105.43) |
|  | upper cumulative sums drifted beyond five standard deviations above a target mean | 23.61(9.84) | 24.92(11.71) | 24.6(8.47) |
|  | lower cumulative sums drifted beyond five standard deviations below a target mean | 45.33(25.55) | 49.07(24.61) | 46.13(24.9) |
|  | index at which the mean of curve changes most significantly | 55.81(8.26) | 55.51(11.03) | 53.33(11.55) |
|  | Area Under the Curve of Power Spectral Density | 3.89(3.17) | 4.75(3.39) | 5.51(4.16) |
|  | Maximum Value of Area Under the Curve of Power Spectral Density | 353.59(291.91) | 438.52(318.23) | 512.41(392.63) |
|  | Occupied bandwidth | 2.14(0.33) | 2.16(0.34) | 2.15(0.36) |
|  | Lower bandwidth frequency bounds | 0(0) | 0(0) | 0(0) |
|  | Upper bandwidth frequency bounds | 2.15(0.33) | 2.16(0.34) | 2.15(0.36) |
|  | Power stored in bandwidth | 3233.56(5937.24) | 4463.91(6329.53) | 6315.39(8180.88) |
|  | Power spectrum | 0.31(0.28) | 0.37(0.28) | 0.41(0.34) |
|  | Max Spectrum Frequencies | 16.41(15.38) | 19.98(16.04) | 22.02(19.03) |
|  | Area Under the Curve of Power Measurment in dB | -Inf | -Inf | -Inf |
|  | Area Under the Curve of Absoulute Value of Power Measurment in dB | Inf(NaN) | Inf(NaN) | Inf(NaN) |
|  | Maximum Value of Power Measurment in dB | -Inf | -Inf | -Inf |
|  | Number of Peaks | 2.77(0.87) | 2.62(0.96) | 2.4(0.91) |
|  | Number of Peaks of absoulte Value | 3.85(0.99) | 3.7(1.14) | 3.55(1.03) |
| Knee Rotation Moment | Maximum Value Druing Mid-Stance | -0.12(0.22) | -0.19(0.25) | -0.26(0.22) |
|  | Minimum Value of the Curve | -1.43(0.6) | -1.48(0.79) | -1.59(0.78) |
|  | Area Under the Curve of Stance Phase | -41.81(19.84) | -45.68(27.78) | -49.53(26.21) |
|  | Area Under the Curve | -41.87(19.76) | -45.66(27.59) | -49.59(26.09) |
|  | Area Under the Curve of Absolute Value During Stance Phase | 42.5(19.14) | 46.59(26.77) | 49.96(25.81) |
|  | Area Under the Curve of Absolute Value | 43.39(19.3) | 47.4(27.12) | 50.9(25.96) |
|  | Root Mean Square | 0.65(0.28) | 0.69(0.39) | 0.74(0.38) |
|  | Mean | -0.42(0.2) | -0.45(0.27) | -0.49(0.26) |
|  | Variance | 0.29(0.25) | 0.35(0.48) | 0.38(0.34) |
|  | Standard Deviation | 0.5(0.21) | 0.52(0.28) | 0.56(0.28) |
|  | Kurtosis | 2.25(0.45) | 2.13(0.52) | 2.12(0.47) |
|  | Peak2RMS | 2.22(0.18) | 2.17(0.22) | 2.16(0.18) |
|  | Maximum - Minimum | 1.5(0.61) | 1.55(0.79) | 1.65(0.78) |
|  |  | 0(0) | 0(0) | 0(0) |
|  |  | 0(0) | 0(0) | 0(0) |
|  | Upper Bound of Autocorrelation | 0.44(0) | 0.44(0) | 0.44(0) |
|  | Lower Bound of Autocorrelation | -0.44(0) | -0.44(0) | -0.44(0) |
|  | Absolute Value of Area Under the Curve During Stance Phase | 41.93(19.57) | 46.08(27.09) | 49.53(26.21) |
|  | Absolute Value of Area Under the Curve | 41.99(19.49) | 46.06(26.93) | 49.59(26.09) |
|  | Root Mean Square of Absolute Value | 0.65(0.28) | 0.69(0.39) | 0.74(0.38) |
|  | Mean of Absolute Value | 0.43(0.19) | 0.47(0.27) | 0.5(0.26) |
|  | Variance of Absolute Value | 0.28(0.24) | 0.34(0.45) | 0.37(0.33) |
|  | Standard Deviation of Absolute Value | 0.49(0.21) | 0.51(0.28) | 0.54(0.28) |
|  | Kurtosis of Absolute Value | 2.29(0.48) | 2.17(0.56) | 2.14(0.51) |
|  | Peak2RMS of Absolute Value | 2.22(0.18) | 2.17(0.22) | 2.16(0.18) |
|  | Absolute Maximum - Absolute Minimum | 1.43(0.6) | 1.48(0.78) | 1.59(0.78) |
|  | Time instants of the mid-reference level crossings | 1.53(0.54) | 1.49(0.63) | 1.64(0.62) |
|  | Mid-reference level | -0.71(0.31) | -0.72(0.4) | -0.78(0.39) |
|  | Minimum distance between signals | 40.97(18.9) | 45.17(26.18) | 48.06(25.33) |
|  | upper cumulative sums drifted beyond five standard deviations above a target mean | 57.36(25.28) | 59.38(22.25) | 56.78(22.7) |
|  | lower cumulative sums drifted beyond five standard deviations below a target mean | 24.55(4.33) | 24.64(6.56) | 24.53(4.88) |
|  | index at which the mean of curve changes most significantly | 58.48(10.37) | 56.88(12.51) | 57.55(10.7) |
|  | Area Under the Curve of Power Spectral Density | 0.29(0.25) | 0.35(0.48) | 0.38(0.34) |
|  | Maximum Value of Area Under the Curve of Power Spectral Density | 23.72(20.38) | 29.56(44.57) | 31.88(28.67) |
|  | Occupied bandwidth | 1.24(0.22) | 1.33(0.3) | 1.34(0.25) |
|  | Lower bandwidth frequency bounds | 0(0) | 0(0) | 0(0) |
|  | Upper bandwidth frequency bounds | 1.24(0.22) | 1.33(0.3) | 1.34(0.25) |
|  | Power stored in bandwidth | 15.98(37.25) | 43.79(343.33) | 29.45(50.19) |
|  | Power spectrum | 0.04(0.03) | 0.04(0.05) | 0.05(0.04) |
|  | Max Spectrum Frequencies | 1.93(1.6) | 2.31(2.76) | 2.57(2.3) |
|  | Area Under the Curve of Power Measurment in dB | -Inf | -Inf | -Inf |
|  | Area Under the Curve of Absoulute Value of Power Measurment in dB | Inf(NaN) | Inf(NaN) | Inf(NaN) |
|  | Maximum Value of Power Measurment in dB | -Inf | -Inf | -Inf |
|  | Number of Peaks | 3.12(1.34) | 3.16(1.73) | 2.93(1.72) |
|  | Number of Peaks of absoulte Value | 4.87(1.58) | 4.81(1.93) | 4.65(1.8) |
| Knee Flexion Angle | Maximum Value Druing Loading Response to Mid-Stance | 21.75(7.78) | 23.36(8.94) | 23.01(8.62) |
|  | Minimum Value during Terminal Stance | 13.83(7.53) | 17.03(9.15) | 17.28(9.29) |
|  | Maximum Value during Initial Swing to Mid-Swing | 62.15(6.89) | 60.19(9.21) | 58.07(12.22) |
|  | Minimum Value druing Terminal Swing | 11.93(7.11) | 14.81(7.62) | 14.84(7) |
|  | Area Under the Curve of Stance Phase | 1190.94(466.24) | 1359.51(559.26) | 1317.87(549.49) |
|  | Area Under the Curve | 2679.18(566.61) | 2808.13(704.4) | 2775.2(743.24) |
|  | Area Under the Curve of Absolute Value During Stance Phase | 1212.08(413.39) | 1370.54(534.41) | 1318.06(549.11) |
|  | Area Under the Curve of Absolute Value | 2701.48(516.77) | 2820.03(680.14) | 2775.39(742.87) |
|  | Root Mean Square | 31.63(4.19) | 32.06(5.64) | 31.4(6.8) |
|  | Mean | 26.65(5.67) | 27.96(7.04) | 27.63(7.42) |
|  | Variance | 279.1(109.53) | 227.72(110.94) | 216.22(116.47) |
|  | Standard Deviation | 16.37(3.27) | 14.53(4.06) | 13.93(4.72) |
|  | Kurtosis | 2.75(0.34) | 2.87(0.52) | 2.82(0.46) |
|  | Peak2RMS | 1.98(0.18) | 1.9(0.26) | 1.86(0.28) |
|  | Maximum - Minimum | 51.4(8.82) | 46.29(11.48) | 44.3(13.34) |
|  | Upper Bound of Autocorrelation | 0.44(0) | 0.44(0) | 0.44(0) |
|  | Lower Bound of Autocorrelation | -0.44(0) | -0.44(0) | -0.44(0) |
|  | Absolute Value of Area Under the Curve During Stance Phase | 1206.95(422.77) | 1363.29(549.93) | 1317.87(549.49) |
|  | Absolute Value of Area Under the Curve | 2679.18(566.61) | 2808.13(704.4) | 2775.2(743.24) |
|  | Root Mean Square of Absolute Value | 31.63(4.19) | 32.06(5.64) | 31.4(6.8) |
|  | Mean of Absolute Value | 26.88(5.17) | 28.08(6.79) | 27.63(7.41) |
|  | Variance of Absolute Value | 271.47(86.12) | 227.32(105.12) | 215.97(116.42) |
|  | Standard Deviation of Absolute Value | 16.22(2.93) | 14.56(3.94) | 13.93(4.72) |
|  | Kurtosis of Absolute Value | 2.76(0.34) | 2.88(0.52) | 2.82(0.46) |
|  | Peak2RMS of Absolute Value | 1.98(0.18) | 1.9(0.26) | 1.86(0.28) |
|  | Absolute Maximum - Absolute Minimum | 51.15(8.13) | 46.29(11.18) | 44.24(13.33) |
|  | Time instants of the mid-reference level crossings | 1.83(0.52) | 1.83(0.49) | 1.87(0.7) |
|  | Mid-reference level | 37.67(5.9) | 38.33(7.22) | 37.08(8.1) |
|  | Minimum distance between signals | 1051.28(248.99) | 950.38(294.79) | 905.99(308.83) |
|  | upper cumulative sums drifted beyond five standard deviations above a target mean | 48.71(20.09) | 46.57(20.52) | 40.98(21.38) |
|  | lower cumulative sums drifted beyond five standard deviations below a target mean | 41.72(28.69) | 39.8(31.11) | 48(37.68) |
|  | index at which the mean of curve changes most significantly | 62.01(4.72) | 63.32(6.22) | 63.07(8.25) |
|  | Area Under the Curve of Power Spectral Density | 276.99(108.54) | 225.94(110) | 214.6(115.55) |
|  | Maximum Value of Area Under the Curve of Power Spectral Density | 17387.43(8622.64) | 14269.29(8091.43) | 13533.96(7796.47) |
|  | Occupied bandwidth | 1.97(0.23) | 1.98(0.26) | 1.94(0.24) |
|  | Lower bandwidth frequency bounds | 0(0) | 0(0) | 0(0) |
|  | Upper bandwidth frequency bounds | 1.97(0.23) | 1.99(0.26) | 1.94(0.24) |
|  | Power stored in bandwidth | 7470965.86(9791956.98) | 5395851.08(5432607.29) | 4869416.29(4037042.65) |
|  | Power spectrum | 12.27(6.12) | 14.72(9.07) | 15.86(10.8) |
|  | Max Spectrum Frequencies | 1013.5(518.87) | 1224.49(755.23) | 1303.57(878.28) |
|  | Area Under the Curve of Power Measurment in dB | -210367.56(17442.73) | -212031.37(15584.67) | -209472.15(24714.63) |
|  | Area Under the Curve of Absoulute Value of Power Measurment in dB | 223568.15(16919.89) | 224772.64(15304.53) | 222622.78(24555.95) |
|  | Maximum Value of Power Measurment in dB | 29.36(2.7) | 29.76(3.7) | 30(3.67) |
|  | Number of Peaks | 2.43(0.61) | 2.76(0.82) | 2.8(0.78) |
|  | Number of Peaks of absoulte Value | 2.46(0.64) | 2.83(0.87) | 2.82(0.8) |
| Knee Power | Maximum Value during Mid-Stance | 3.48(3.02) | 2.79(3.02) | 2.24(2.38) |
|  | Maximum Value during Mid-Stance to Terminal Stance | -0.27(0.69) | -0.57(1.19) | -0.42(0.87) |
|  | Maximum Value during Terminal Stance to Pre-Swing | 0.97(1.92) | 0.85(1.01) | 0.95(1.65) |
|  | Minimum Value during Pre-Swing to Initial Swing | -6.3(3.75) | -5.28(3.64) | -5.81(3.52) |
|  | Area Under the Curve of Stance Phase | -36.15(45.42) | -39.06(42.1) | -41.75(47.27) |
|  | Area Under the Curve | -128.72(69.83) | -113.58(70.07) | -123.47(74.93) |
|  | Area Under the Curve of Absolute Value During Stance Phase | 128.73(81.17) | 107.76(77.92) | 101.29(64.43) |
|  | Area Under the Curve of Absolute Value | 225.19(110.21) | 184.85(109.04) | 186.03(97.47) |
|  | Root Mean Square | 3.26(1.55) | 2.66(1.5) | 2.73(1.39) |
|  | Mean | -1.27(0.69) | -1.12(0.69) | -1.22(0.74) |
|  | Variance | 11.06(12.26) | 7.61(8.68) | 7.37(6.7) |
|  | Standard Deviation | 2.98(1.48) | 2.39(1.37) | 2.43(1.23) |
|  | Kurtosis | 3.44(1.09) | 3.52(1.05) | 3.52(1.39) |
|  | Peak2RMS | 2.74(0.37) | 2.74(0.39) | 2.74(0.4) |
|  | Maximum - Minimum | 12.57(6.57) | 10.2(6.11) | 10.13(5.32) |
|  | Upper Bound of Autocorrelation | 0.42(0.01) | 0.42(0.01) | 0.42(0.01) |
|  | Lower Bound of Autocorrelation | -0.42(0.01) | -0.42(0.01) | -0.42(0.01) |
|  | Absolute Value of Area Under the Curve During Stance Phase | 43.52(38.37) | 42.35(38.77) | 46.82(42.14) |
|  | Absolute Value of Area Under the Curve | 128.95(69.42) | 113.58(70.07) | 123.54(74.81) |
|  | Root Mean Square of Absolute Value | 3.26(1.55) | 2.66(1.5) | 2.73(1.39) |
|  | Mean of Absolute Value | 2.24(1.09) | 1.84(1.08) | 1.85(0.97) |
|  | Variance of Absolute Value | 6.91(6.88) | 4.78(5.28) | 5.04(4.71) |
|  | Standard Deviation of Absolute Value | 2.37(1.14) | 1.91(1.06) | 2(1.03) |
|  | Kurtosis of Absolute Value | 3.85(1.52) | 3.9(1.6) | 3.87(1.64) |
|  | Peak2RMS of Absolute Value | 2.74(0.37) | 2.74(0.39) | 2.74(0.4) |
|  | Absolute Maximum - Absolute Minimum | 8.88(4.31) | 7.12(3.86) | 7.44(3.93) |
|  | Time instants of the mid-reference level crossings | 3.62(1.85) | 3.49(2.05) | 3.22(1.71) |
|  | Mid-reference level | -3(2.07) | -2.33(1.77) | -2.74(2.15) |
|  | Minimum distance between signals | 109.66(64.64) | 89.72(59.35) | 90.91(49.25) |
|  | upper cumulative sums drifted beyond five standard deviations above a target mean | 13.29(16.42) | 14.52(15.55) | 15.73(16.69) |
|  | lower cumulative sums drifted beyond five standard deviations below a target mean | 20.59(19.21) | 25.31(22.31) | 25.8(20.18) |
|  | index at which the mean of curve changes most significantly | 57.56(21.23) | 56.24(21.22) | 53.76(17.93) |
|  | Area Under the Curve of Power Spectral Density | 10.96(12.18) | 7.53(8.6) | 7.3(6.63) |
|  | Maximum Value of Area Under the Curve of Power Spectral Density | 430.42(462.82) | 294.47(329.04) | 290.06(257.06) |
|  | Occupied bandwidth | 2.09(0.46) | 2.03(0.49) | 2.07(0.49) |
|  | Lower bandwidth frequency bounds | 0(0) | 0(0) | 0(0) |
|  | Upper bandwidth frequency bounds | 2.09(0.46) | 2.03(0.49) | 2.07(0.49) |
|  | Power stored in bandwidth | 11723.6(43042.88) | 5766.42(16219.29) | 4423.47(7239.85) |
|  | Power spectrum | 0.29(0.41) | 0.22(0.36) | 0.24(0.3) |
|  | Max Spectrum Frequencies | 8.5(12.27) | 6.72(10.89) | 7.21(9.15) |
|  | Area Under the Curve of Power Measurment in dB | #NAME? | #NAME? | #NAME? |
|  | Area Under the Curve of Absoulute Value of Power Measurment in dB | Inf(NaN) | Inf(NaN) | Inf(NaN) |
|  | Maximum Value of Power Measurment in dB | #NAME? | #NAME? | #NAME? |
|  | Number of Peaks | 4.07(1.17) | 4.32(1.47) | 4.49(1.39) |
|  | Number of Peaks of absoulte Value | 7.21(1.78) | 7.24(2.2) | 7.76(2.04) |
|  | Maximum Value during Loading Response | -4.4(4.14) | -3.6(3.5) | -2.89(3.06) |
|  | Maximum Value During Mid-Stance | 3.48(3.02) | 2.79(3.02) | 2.24(2.38) |
|  | Minimum Value during Pre-Swing to Initial Swing | -6.3(3.75) | -5.28(3.64) | -5.81(3.52) |
|  | Maximum Value during Mid-Swing | 0.14(0.25) | 0.08(0.22) | 0.12(0.25) |
|  | Maximum Value during Terminal Swing | -7.47(3.65) | -5.8(3.36) | -6.25(3.58) |
| Knee Varus Angle | Maximum Value during Mid-Stance | 5.36(4.55) | 8.81(6.06) | 9.34(6.35) |
|  | Minimum Value during Mid-Stance to Terminal Stance | 4.11(4.53) | 7.34(6.04) | 8.07(6.63) |
|  | Maximum Value during Terminal Stance | 5.09(4.4) | 8.46(6.01) | 8.95(6.53) |
|  | Minimum Value during Pre-Swing to Initial Swing | -1.74(4.84) | 0.95(5.52) | 2.18(6.13) |
|  | Area Under the Curve of Stance Phase | 274.47(277.05) | 490.8(377.33) | 517.3(401.42) |
|  | Area Under the Curve | 334.36(420.53) | 641.28(530.54) | 710.69(595.35) |
|  | Area Under the Curve of Absolute Value During Stance Phase | 307.24(235.14) | 534.12(291.53) | 565.71(314.49) |
|  | Area Under the Curve of Absolute Value | 468.03(305.22) | 734.22(387.58) | 811.47(435.41) |
|  | Root Mean Square (RMS) | 5.18(3.03) | 7.77(3.9) | 8.64(4.28) |
|  | Mean | 3.24(4.2) | 6.24(5.3) | 6.97(5.94) |
|  | Variance | 7.14(6.12) | 8.4(7.87) | 6.75(6.3) |
|  | Standard Deviation | 2.46(1.06) | 2.64(1.2) | 2.39(1.04) |
|  | Kurtosis | 3.31(0.94) | 3.21(0.95) | 2.83(0.73) |
|  | Peak2RMS | 1.73(0.49) | 1.42(0.34) | 1.45(0.39) |
|  | Maximum - Minimum | 8.8(3.49) | 9.29(3.72) | 8.37(3.39) |
|  | Upper Bound of Autocorrelation | 0.43(0.01) | 0.44(0.01) | 0.43(0.01) |
|  | Lower Bound of Autocorrelation | -0.43(0.01) | -0.44(0.01) | -0.43(0.01) |
|  | Absolute Value of Area Under the Curve During Stance Phase | 302.03(239.71) | 531.36(295.59) | 562.57(319.35) |
|  | Absolute Value of Area Under the Curve | 399.64(348.81) | 706.91(412.45) | 794.27(454.28) |
|  | Root Mean Square of Absolute Value | 5.1(3.03) | 7.75(3.9) | 8.48(4.28) |
|  | Mean of Absolute Value | 4.68(3.05) | 7.34(3.88) | 8.11(4.35) |
|  | Variance of Absolute Value | 4.03(3.96) | 6.45(6.44) | 5.63(5.39) |
|  | Standard Deviation of Absolute Value | 1.82(0.85) | 2.29(1.09) | 2.18(0.95) |
|  | Kurtosis of Absolute Value | 3.23(1.05) | 3.03(0.96) | 2.81(0.96) |
|  | Peak2RMS of Absolute Value | 1.73(0.49) | 1.43(0.34) | 1.45(0.39) |
|  | Absolute Maximum - Absolute Minimum | 6.83(2.77) | 8.14(3.36) | 7.64(2.97) |
|  | Time instants of the mid-reference level crossings | 2.03(1.28) | 1.67(0.83) | 1.75(1) |
|  | Mid-reference level | 308.36(239.71) | 534.6(295.59) | 573.58(319.35) |
|  | Minimum distance between signals | 145.69(80.8) | 168.81(102.37) | 154.01(95.64) |
|  | upper cumulative sums drifted beyond five standard deviations above a target mean | 31.32(26.28) | 26.12(24.25) | 25.16(21.67) |
|  | lower cumulative sums drifted beyond five standard deviations below a target mean | 41.46(22.67) | 44.71(20.66) | 46.82(19.71) |
|  | index at which the mean of curve changes most significantly | 62.12(11.96) | 61.55(9.4) | 64.04(10.83) |
|  | Area Under the Curve of Power Spectral Density | 7.09(6.08) | 8.34(7.8) | 6.7(6.25) |
|  | Maximum Value of Area Under the Curve of Power Spectral Density | 443.86(392.26) | 577.51(596.78) | 465.04(479.79) |
|  | Occupied bandwidth | 1.72(0.4) | 1.9(0.41) | 1.89(0.42) |
|  | Lower bandwidth frequency bounds | 0(0) | 0(0) | 0(0) |
|  | Upper bandwidth frequency bounds | 1.73(0.4) | 1.9(0.41) | 1.89(0.42) |
|  | Power stored in bandwidth | 6826.89(12809.7) | 12264.39(25891.07) | 7810.71(17175.7) |
|  | Area Under the Curve of Power Spectrum | 0.94(1.26) | 2.19(2.31) | 2.65(2.38) |
|  | Max Spectrum Frequencies | 61.59(89.95) | 151(167.14) | 183.2(172.92) |
|  | Area Under the Curve of Power Measurment in dB | -229139.15(20616.22) | -228270.48(17556.33) | -227383.27(26438.9) |
|  | Area Under the Curve of Absoulute Value of Power Measurment in dB | 234186.91(20382.02) | 235834.65(17421.15) | 235077.93(26052.17) |
|  | Maximum Value of Power Measurment in dB | 13.22(7.23) | 18.8(6.37) | 19.46(6.65) |
|  | Number of Peaks | 4.01(1.18) | 4.01(1.22) | 4.11(1.07) |
|  | Number of Peaks of absoulte Value | 4.77(1.27) | 4.48(1.3) | 4.44(1.13) |
|  | Minimum Value during Initial Swing to Mid-Swing | -2.39(4.89) | 0.14(5.18) | 1.62(6.12) |
|  | Maximum Value during Terminal Swing | 5.39(4.29) | 7.88(5.03) | 8.5(5.47) |
|  | Minimum Value during Loading Response | 3.12(4.14) | 6.18(5.52) | 6.4(5.71) |
|  | Maximum Value during Loading Response to Mid-Stance | 5.21(4.54) | 8.62(5.71) | 9.2(6.11) |
| Ankle Dorsiflexion Moment | Maximum value during initial Swing | -0.3(0.11) | -0.23(0.14) | -0.26(0.11) |
|  | Maximum Value of the Curve | 10.73(3.46) | 9.39(3.21) | 9.72(2.69) |
|  | Minimum Value during Pre-Swing to Initial Swing | -0.3(0.11) | -0.23(0.14) | -0.26(0.11) |
|  | Area Under the Curve of Stance Phase | 294.31(105.72) | 277.24(102.91) | 283.78(95.94) |
|  | Area Under the Curve | 292.07(105.29) | 275.39(102.35) | 282.34(95.05) |
|  | Area Under the Curve of Absolute Value During Stance Phase | 304.3(105.72) | 282.71(102.52) | 289.1(94.59) |
|  | Area Under the Curve of Absolute Value | 308.62(106.9) | 286.36(103.57) | 293.7(95.07) |
|  | Root Mean Square | 4.75(1.59) | 4.29(1.5) | 4.41(1.35) |
|  | Mean | 2.89(1.04) | 2.73(1.01) | 2.79(0.94) |
|  | Variance | 15.76(10.37) | 12.33(8.27) | 12.67(6.72) |
|  | Standard Deviation | 3.77(1.24) | 3.32(1.15) | 3.42(1.01) |
|  | Kurtosis | 2.36(0.49) | 2.18(0.46) | 2.22(0.4) |
|  | Peak2RMS | 2.28(0.19) | 2.2(0.2) | 2.23(0.16) |
|  | Maximum - Minimum | 11.38(3.68) | 9.76(3.36) | 10.28(2.81) |
|  | Upper Bound of Autocorrelation | 0.44(0) | 0.44(0) | 0.44(0) |
|  | Lower Bound of Autocorrelation | -0.44(0) | -0.44(0) | -0.44(0) |
|  | Absolute Value of Area Under the Curve During Stance Phase | 294.31(105.72) | 277.24(102.91) | 283.78(95.94) |
|  | Absolute Value of Area Under the Curve | 292.07(105.29) | 275.39(102.35) | 282.34(95.05) |
|  | Root Mean Square of Absolute Value | 4.75(1.59) | 4.29(1.5) | 4.41(1.35) |
|  | Mean of Absolute Value | 3.06(1.06) | 2.84(1.03) | 2.91(0.94) |
|  | Variance of Absolute Value | 14.73(9.86) | 11.69(7.9) | 12.01(6.48) |
|  | Standard Deviation of Absolute Value | 3.64(1.21) | 3.23(1.13) | 3.32(0.99) |
|  | Kurtosis of Absolute Value | 2.44(0.53) | 2.24(0.5) | 2.28(0.44) |
|  | Peak2RMS of Absolute Value | 2.28(0.19) | 2.2(0.2) | 2.23(0.16) |
|  | Absolute Maximum - Absolute Minimum | 10.73(3.46) | 9.38(3.21) | 9.71(2.69) |
|  | Time instants of the mid-reference level crossings | 1.93(0.33) | 1.83(0.46) | 1.8(0.45) |
|  | Mid-reference level | 4.99(1.87) | 4.41(1.71) | 4.51(1.57) |
|  | Minimum distance between signals | 301.06(103.74) | 277.66(100.62) | 283.1(92.23) |
|  | upper cumulative sums drifted beyond five standard deviations above a target mean | 24.52(3.5) | 24.05(4.75) | 24.65(3.41) |
|  | lower cumulative sums drifted beyond five standard deviations below a target mean | 40.12(38.29) | 55.23(33.67) | 53.56(32.64) |
|  | index at which the mean of curve changes most significantly | 61.37(9.79) | 60.42(12.31) | 60.96(9.69) |
|  | Area Under the Curve of Power Spectral Density | 15.74(10.34) | 12.32(8.25) | 12.65(6.71) |
|  | Maximum Value of Area Under the Curve of Power Spectral Density | 1294.03(861.31) | 1029.92(697.69) | 1052.1(577.46) |
|  | Occupied bandwidth | 1.22(0.08) | 1.24(0.09) | 1.25(0.12) |
|  | Lower bandwidth frequency bounds | 0(0) | 0(0) | 0(0) |
|  | Upper bandwidth frequency bounds | 1.22(0.08) | 1.25(0.09) | 1.25(0.12) |
|  | Power stored in bandwidth | 39901.35(79688.82) | 25169.86(54975.91) | 23572.86(24103.8) |
|  | Power spectrum | 2.1(1.32) | 1.66(1.07) | 1.7(0.91) |
|  | Max Spectrum Frequencies | 107.82(67.46) | 87.45(55.24) | 88.96(49.79) |
|  | Area Under the Curve of Power Measurment in dB | -Inf | -Inf | -Inf |
|  | Area Under the Curve of Absoulute Value of Power Measurment in dB | Inf(NaN) | Inf(NaN) | Inf(NaN) |
|  | Maximum Value of Power Measurment in dB | -Inf | -Inf | -Inf |
|  | Number of Peaks | 2.84(0.69) | 2.72(0.76) | 2.71(0.79) |
|  | Number of Peaks of absoulte Value | 4.23(1.01) | 3.82(1.12) | 3.82(1.02) |
| Ankle Varus Moment | Maximum Value during Loading Response to Mid-Stance | -13.61(7.01) | -12.69(7.08) | -12.66(7.84) |
|  | Minimum Value during Terminal Stance to Pre-Swing | -10.36(7.26) | -8.58(8.15) | -8.32(8.58) |
|  | Maximum Value during Mid-Swing | -21.66(7.22) | -20.57(8.08) | -21.22(7.05) |
|  | Minimum Value during Terminal Swing | 0.73(0.53) | 0.67(0.52) | 0.68(0.52) |
|  | Area Under the Curve of Stance Phase | 0.52(0.53) | 0.43(0.51) | 0.45(0.49) |
|  | Area Under the Curve | 1.2(0.69) | 0.98(0.64) | 0.96(0.58) |
|  | Area Under the Curve of Absolute Value During Stance Phase | 0(0.01) | 0(0.01) | 0(0.02) |
|  | Area Under the Curve of Absolute Value | 31.81(26.27) | 27.65(25.15) | 30.57(25.52) |
|  | Root Mean Square | 31.96(26.17) | 27.73(24.99) | 30.77(25.37) |
|  | Mean | 37.71(20.13) | 33.21(20.33) | 33.65(22.93) |
|  | Variance | 38.64(20.43) | 33.91(20.56) | 34.46(23.11) |
|  | Standard Deviation | 0.56(0.29) | 0.48(0.28) | 0.48(0.3) |
|  | Kurtosis | 0.32(0.26) | 0.27(0.25) | 0.3(0.25) |
|  | Peak2RMS | 0.23(0.21) | 0.18(0.23) | 0.17(0.18) |
|  | Maximum - Minimum | 0.43(0.21) | 0.37(0.2) | 0.35(0.2) |
|  | Upper Bound of Autocorrelation | 2.47(0.88) | 2.33(0.88) | 2.35(0.94) |
|  | Lower Bound of Autocorrelation | 2.28(0.32) | 2.19(0.36) | 2.19(0.38) |
|  | Absolute Value of Area Under the Curve During Stance Phase | 1.4(0.65) | 1.17(0.63) | 1.11(0.57) |
|  | Absolute Value of Area Under the Curve | 0.44(0) | 0.44(0) | 0.44(0) |
|  | Root Mean Square of Absolute Value | -0.44(0) | -0.44(0) | -0.44(0) |
|  | Mean of Absolute Value | 34.83(22.07) | 30.46(21.64) | 31.82(23.91) |
|  | Variance of Absolute Value | 34.88(22.1) | 30.48(21.52) | 31.93(23.86) |
|  | Standard Deviation of Absolute Value | 0.38(0.2) | 0.34(0.2) | 0.34(0.23) |
|  | Kurtosis of Absolute Value | 0.21(0.21) | 0.16(0.21) | 0.15(0.17) |
|  | Peak2RMS of Absolute Value | 0.4(0.21) | 0.34(0.2) | 0.33(0.2) |
|  | Absolute Maximum - Absolute Minimum | 2.52(0.96) | 2.35(0.97) | 2.37(1.07) |
|  | Time instants of the mid-reference level crossings | 2.28(0.32) | 2.19(0.36) | 2.19(0.38) |
|  | Mid-reference level | 1.25(0.64) | 1.03(0.6) | 1(0.56) |
|  | Minimum distance between signals | 1.78(0.75) | 1.66(0.64) | 1.58(0.69) |
|  | upper cumulative sums drifted beyond five standard deviations above a target mean | 0.44(0.37) | 0.38(0.31) | 0.39(0.31) |
|  | lower cumulative sums drifted beyond five standard deviations below a target mean | 35.66(19.11) | 31.59(19.79) | 31.11(21.68) |
|  | index at which the mean of curve changes most significantly | 27.96(10.69) | 26.85(11.64) | 27.02(12.36) |
|  | Area Under the Curve of Power Spectral Density | 28.82(29.85) | 30.28(30.25) | 41.42(27.03) |
|  | Maximum Value of Area Under the Curve of Power Spectral Density | 53.86(16.52) | 52.45(17.34) | 56.09(15.46) |
|  | Occupied bandwidth | 0.23(0.21) | 0.18(0.23) | 0.17(0.18) |
|  | Lower bandwidth frequency bounds | 17.3(16.8) | 13.79(18.49) | 13.3(15.75) |
|  | Upper bandwidth frequency bounds | 1.45(0.5) | 1.6(0.54) | 1.65(0.58) |
|  | Power stored in bandwidth | 0(0) | 0(0) | 0(0) |
|  | Power spectrum | 1.45(0.5) | 1.6(0.54) | 1.65(0.58) |
|  | Max Spectrum Frequencies | 9.73(19.82) | 8.71(41.21) | 6.74(13.77) |
|  | Area Under the Curve of Power Measurment in dB | 0.03(0.03) | 0.02(0.03) | 0.02(0.02) |
|  | Area Under the Curve of Absoulute Value of Power Measurment in dB | 1.5(1.65) | 1.11(1.55) | 1.06(1.2) |
|  | Maximum Value of Power Measurment in dB | -Inf | -Inf | -Inf |
|  | Number of Peaks | Inf(NaN) | Inf(NaN) | Inf(NaN) |
|  | Number of Peaks of absoulte Value | -Inf | -Inf | -Inf |
| Ankle Flexion Angle | Maximum Value during Loading Response to Mid-Stance | 13.35(4.17) | 14(5.42) | 15.98(6.25) |
|  | Minimum Value during Terminal Stance | 11.17(4.12) | 11.57(5.3) | 13.62(6.45) |
|  | Maximum Value during Pre-Swing to Initial Swing | 13.12(4.2) | 13.35(5.34) | 15.72(6.1) |
|  | Minimum Value during Terminal Swing | 15.55(3.5) | 16.4(4.24) | 16.86(4.21) |
|  | Area Under the Curve of Stance Phase | 13.68(3.56) | 14.61(4.45) | 14.77(4.21) |
|  | Area Under the Curve | 18.65(3.48) | 19.66(3.84) | 19.99(4.03) |
|  | Area Under the Curve of Absolute Value During Stance Phase | 3.72(3.54) | 4.6(3.6) | 5.39(3.95) |
|  | Area Under the Curve of Absolute Value | 637.19(233.24) | 720.31(255.22) | 697.53(245.79) |
|  | Root Mean Square | 782.01(328.75) | 920.42(339.27) | 942.62(337.76) |
|  | Mean | 684.69(174.9) | 756(212.58) | 731.84(205.99) |
|  | Variance | 924.49(215.61) | 1024.58(255.5) | 1028.03(255.01) |
|  | Standard Deviation | 10.77(2.18) | 11.69(2.57) | 11.8(2.64) |
|  | Kurtosis | 7.78(3.28) | 9.16(3.38) | 9.38(3.37) |
|  | Peak2RMS | 50.01(18.63) | 48.45(18.28) | 47.27(16.49) |
|  | Maximum - Minimum | 6.96(1.24) | 6.83(1.34) | 6.76(1.25) |
|  | Upper Bound of Autocorrelation | 2.23(0.41) | 2.23(0.47) | 2.15(0.38) |
|  | Lower Bound of Autocorrelation | 1.78(0.17) | 1.71(0.16) | 1.72(0.15) |
|  | Absolute Value of Area Under the Curve During Stance Phase | 24.8(5.4) | 24.3(5.4) | 23.85(4.81) |
|  | Absolute Value of Area Under the Curve | 0.43(0) | 0.43(0) | 0.43(0) |
|  | Root Mean Square of Absolute Value | -0.43(0) | -0.43(0) | -0.43(0) |
|  | Mean of Absolute Value | 643.37(215.49) | 720.31(255.22) | 697.53(245.79) |
|  | Variance of Absolute Value | 789.99(308.94) | 920.42(339.27) | 942.92(336.92) |
|  | Standard Deviation of Absolute Value | 9.19(2.15) | 10.19(2.55) | 10.23(2.55) |
|  | Kurtosis of Absolute Value | 31.88(10.87) | 33.19(12.13) | 35.3(14.09) |
|  | Peak2RMS of Absolute Value | 5.56(0.97) | 5.66(1.08) | 5.82(1.22) |
|  | Absolute Maximum - Absolute Minimum | 1.98(0.28) | 1.96(0.24) | 1.94(0.19) |
|  | Time instants of the mid-reference level crossings | 1.78(0.17) | 1.71(0.16) | 1.72(0.15) |
|  | Mid-reference level | 18.5(3.33) | 19(3.49) | 19.47(4.02) |
|  | Minimum distance between signals | 2.13(0.7) | 2.18(0.88) | 2.33(1.02) |
|  | upper cumulative sums drifted beyond five standard deviations above a target mean | 8.12(5.73) | 8.87(5.12) | 9.09(4.88) |
|  | lower cumulative sums drifted beyond five standard deviations below a target mean | 283.67(124.64) | 290.95(122.06) | 243.33(142.49) |
|  | index at which the mean of curve changes most significantly | 25.32(0.64) | 25.38(0.76) | 25.55(1.09) |
|  | Area Under the Curve of Power Spectral Density | 19.35(22.1) | 17.85(21.53) | 19(23.2) |
|  | Maximum Value of Area Under the Curve of Power Spectral Density | 47.74(20.05) | 44.92(21) | 36.05(19.14) |
|  | Occupied bandwidth | 49.81(18.53) | 48.26(18.2) | 47.07(16.43) |
|  | Lower bandwidth frequency bounds | 3121.84(1212.75) | 3122.97(1178.74) | 2935.57(1244.52) |
|  | Upper bandwidth frequency bounds | 1.24(0.25) | 1.33(0.36) | 1.36(0.34) |
|  | Power stored in bandwidth | 0(0) | 0(0) | 0(0) |
|  | Power spectrum | 1.24(0.25) | 1.33(0.36) | 1.36(0.34) |
|  | Max Spectrum Frequencies | 216297.52(210359.19) | 208690.81(152924.06) | 192872.39(143392.51) |
|  | Area Under the Curve of Power Measurment in dB | 6.95(2.66) | 7.88(3.3) | 8.04(3.37) |
|  | Area Under the Curve of Absoulute Value of Power Measurment in dB | 381.85(195.32) | 458.14(231.96) | 463.27(229.51) |
|  | Maximum Value of Power Measurment in dB | -200643.42(17871.47) | -205653.08(18302.32) | -200523.36(21686.28) |
|  | Number of Peaks | 215170.3(16972.54) | 219996.73(17258.55) | 215358.64(20704.27) |
|  | Number of Peaks of absoulte Value | 25.17(2.58) | 25.94(2.62) | 26.02(2.55) |
| Ankle Power | Maximum Value during Mid-Stance | -0.07(0.87) | -0.39(0.86) | -0.57(1.03) |
|  | Maximum Value during Mid-Stance to Terminal Stance | -4.8(2.23) | -3.99(2.03) | -4.64(1.92) |
|  | Maximum Value during Terminal Stance to Pre-Swing | 14.86(7.6) | 11.68(7.7) | 13.31(6.03) |
|  | Minimum Value during Pre-Swing to Initial Swing | -1.86(2.37) | -1.86(2.06) | -1.83(2.41) |
|  | Area Under the Curve of Stance Phase | 12.58(48.88) | -1.28(60.19) | -11.33(45.74) |
|  | Area Under the Curve | 18.13(50.04) | 3.27(61.98) | -4.65(47.12) |
|  | Area Under the Curve of Absolute Value During Stance Phase | 249.48(103.15) | 214.86(100.75) | 243.44(91.08) |
|  | Area Under the Curve of Absolute Value | 256.57(106.49) | 220.6(102.92) | 251.27(92.55) |
|  | Root Mean Square | 4.48(1.92) | 3.72(1.88) | 4.15(1.54) |
|  | Mean | 0.18(0.5) | 0.03(0.61) | -0.05(0.47) |
|  | Variance | 23.68(20.08) | 17.14(18.4) | 19.56(13.5) |
|  | Standard Deviation | 4.47(1.92) | 3.69(1.89) | 4.15(1.56) |
|  | Kurtosis | 6.73(1.14) | 6.01(1.48) | 6.12(1.27) |
|  | Peak2RMS | 3.44(0.32) | 3.24(0.43) | 3.28(0.34) |
|  | Maximum - Minimum | 20.98(9.02) | 16.62(8.73) | 19.15(6.99) |
|  | Upper Bound of Autocorrelation | 0.43(0) | 0.43(0) | 0.43(0) |
|  | Lower Bound of Autocorrelation | -0.43(0.0031) | -0.43(0.0030) | -0.43(0.0032) |
|  | Absolute Value of Area Under the Curve During Stance Phase | 39.9(30.76) | 46.09(38.58) | 34.99(31.24) |
|  | Absolute Value of Area Under the Curve | 41.54(33.14) | 47.37(39.97) | 35.53(30.94) |
|  | Root Mean Square of Absolute Value | 4.48(1.92) | 3.72(1.88) | 4.15(1.54) |
|  | Mean of Absolute Value | 2.54(1.05) | 2.19(1.02) | 2.49(0.92) |
|  | Variance of Absolute Value | 16.32(13.83) | 11.66(13.07) | 12.69(8.82) |
|  | Standard Deviation of Absolute Value | 3.7(1.63) | 3.01(1.61) | 3.33(1.27) |
|  | Kurtosis of Absolute Value | 6.61(1.68) | 5.81(2.01) | 5.83(1.74) |
|  | Peak2RMS of Absolute Value | 3.44(0.32) | 3.24(0.43) | 3.27(0.34) |
|  | Absolute Maximum - Absolute Minimum | 15.64(7.14) | 12.44(7.09) | 13.79(5.71) |
|  | Time instants of the mid-reference level crossings | 1.97(0.29) | 1.95(0.43) | 1.96(0.27) |
|  | Mid-reference level | 4.62(3.11) | 3.9(3.76) | 4.35(3.07) |
|  | Minimum distance between signals | 249.14(103.08) | 215.28(101.35) | 248.1(92.3) |
|  | upper cumulative sums drifted beyond five standard deviations above a target mean | 32.77(21.58) | 32.64(23.1) | 30.84(22.63) |
|  | lower cumulative sums drifted beyond five standard deviations below a target mean | 24.54(5.69) | 24.44(6.55) | 25.13(5.36) |
|  | index at which the mean of curve changes most significantly | 50.21(8.02) | 49.99(10.1) | 50.02(7.49) |
|  | Area Under the Curve of Power Spectral Density | 23.52(19.94) | 17.03(18.28) | 19.43(13.42) |
|  | Maximum Value of Area Under the Curve of Power Spectral Density | 832.04(768.36) | 658.83(672.78) | 759.58(561.21) |
|  | Occupied Bandwidth | 0.89(0.25) | 1.09(0.35) | 0.98(0.27) |
|  | Lower bandwidth frequency bounds | 0(0) | 0(0) | 0(0) |
|  | Upper bandwidth frequency bounds | 0.89(0.25) | 1.09(0.35) | 0.98(0.27) |
|  | Power stored in bandwidth | 41952.09(106083.1) | 27520.61(98185.39) | 25742.52(35104.51) |
|  | Power spectrum | 1.88(1.76) | 1.35(1.71) | 1.45(1.02) |
|  | Max Spectrum Frequencies | 48.11(43.07) | 34.63(40.2) | 37.85(26.44) |
|  | Area Under the Curve of Power Measurment in dB | -Inf | -Inf | -Inf |
|  | Area Under the Curve of Absoulute Value of Power Measurment in dB | Inf(NaN) | Inf(NaN) | Inf(NaN) |
|  | Maximum Value of Power Measurment in dB | -Inf | -Inf | -Inf |
|  | Number of Peaks | 4.54(1.04) | 4.69(1.33) | 4.44(1.01) |
|  | Number of Peaks of absoulte Value | 7.34(1.49) | 6.94(1.76) | 6.64(1.52) |
|  | Maximum Value during Terminal Stance | 14.86(7.6) | 11.67(7.7) | 13.31(6.03) |
| Foot Progression Moment | Maximum Value during Loading Response to Mid-Stance | -11.85(6.64) | -11.21(6.77) | -10.96(7.27) |
|  | Minimum Value during Terminal Stance to Pre-Swing | -13.45(6.93) | -12.57(7.02) | -12.45(7.69) |
|  | Maximum Value during Pre-swing to Mid-Swing | -10.04(7.04) | -8.27(7.96) | -8.15(8.34) |
|  | Area Under the Curve of Stance Phase | -21.18(7.19) | -20.35(8.02) | -20.81(7.01) |
|  | Area Under the Curve | -814.07(443.02) | -776.1(464.73) | -738.81(500.94) |
|  | Area Under the Curve of Absolute Value During Stance Phase | -1436.02(649.84) | -1339.77(683.77) | -1338.84(715.62) |
|  | Area Under the Curve of Absolute Value | 818.25(435.49) | 786.28(448.33) | 758.48(470.4) |
|  | Root Mean Square | 1442.34(638.03) | 1357.06(654.5) | 1366.23(663.98) |
|  | Mean | 15(6.19) | 14.18(6.36) | 14.38(6.33) |
|  | Variance | -14.38(6.49) | -13.41(6.83) | -13.4(7.16) |
|  | Standard Deviation | 14.64(11.74) | 15.2(16.01) | 16.25(10.76) |
|  | Kurtosis | 3.56(1.41) | 3.54(1.63) | 3.8(1.36) |
|  | Peak2RMS | 2.96(0.86) | 3.07(0.96) | 3.06(0.95) |
|  | Maximum - Minimum | 1.57(0.29) | 1.57(0.34) | 1.62(0.36) |
|  | Upper Bound of Autocorrelation | 12.53(4.42) | 12.73(4.98) | 13.74(4.57) |
|  | Lower Bound of Autocorrelation | 0.43(0.01) | 0.43(0.01) | 0.43(0.01) |
|  | Absolute Value of Area Under the Curve During Stance Phase | -0.43(0.01) | -0.43(0.01) | -0.43(0.01) |
|  | Absolute Value of Area Under the Curve | 816.94(437.66) | 782.65(453.55) | 757.25(472.07) |
|  | Root Mean Square of Absolute Value | 1436.56(648.63) | 1345.31(672.75) | 1361.15(671.39) |
|  | Mean of Absolute Value | 14.44(6.38) | 13.59(6.54) | 13.68(6.64) |
|  | Variance of Absolute Value | 14.33(11.64) | 14.43(15.95) | 15.83(10.54) |
|  | Standard Deviation of Absolute Value | 3.51(1.42) | 3.43(1.63) | 3.75(1.34) |
|  | Kurtosis of Absolute Value | 2.96(0.86) | 3.03(0.97) | 3.04(0.94) |
|  | Peak2RMS of Absolute Value | 1.57(0.29) | 1.57(0.34) | 1.62(0.36) |
|  | Absolute Maximum - Absolute Minimum | 12.29(4.38) | 12.18(4.88) | 13.43(4.38) |
|  | Time instants of the mid-reference level crossings | 1.46(0.96) | 1.56(0.96) | 1.35(0.64) |
|  | Mid-reference level | -16.34(6.72) | -15.04(7.4) | -15.51(6.99) |
|  | Minimum distance between signals | 239.24(115.37) | 241.28(128.84) | 256.97(118.78) |
|  | upper cumulative sums drifted beyond five standard deviations above a target mean | 32.86(24.41) | 38.31(22.32) | 34.45(21.5) |
|  | lower cumulative sums drifted beyond five standard deviations below a target mean | 60.32(24.15) | 59.31(27.69) | 57.98(24.04) |
|  | index at which the mean of curve changes most significantly | 72.25(10.1) | 72.92(11.02) | 72.42(9.06) |
|  | Area Under the Curve of Power Spectral Density | 14.49(11.59) | 15.05(15.83) | 16.09(10.65) |
|  | Maximum Value of Area Under the Curve of Power Spectral Density | 928.61(805.47) | 948.98(1158.26) | 992.13(719.01) |
|  | Occupied bandwidth | 2.28(0.51) | 2.11(0.57) | 2.24(0.51) |
|  | Lower bandwidth frequency bounds | 0(0) | 0(0) | 0(0) |
|  | Upper bandwidth frequency bounds | 2.28(0.51) | 2.11(0.57) | 2.24(0.51) |
|  | Power stored in bandwidth | 30772.46(57706.77) | 44286.16(183755.65) | 31169.7(39409.32) |
|  | Power spectrum | 5.44(5.45) | 4.94(5.17) | 5.1(7.07) |
|  | Max Spectrum Frequencies | 418.67(416.85) | 376.68(395.75) | 387.95(541.94) |
|  | Area Under the Curve of Power Measurment in dB | -229820.3(16799.25) | -232470.84(17287.88) | -227695.05(20058.12) |
|  | Area Under the Curve of Absoulute Value of Power Measurment in dB | 239077.4(17032.97) | 241510.88(17555.15) | 236902.77(19633.87) |
|  | Maximum Value of Power Measurment in dB | 23.56(6.14) | 22.52(6.81) | 22.71(6.54) |
|  | Number of Peaks | 3.09(0.94) | 3.03(1.1) | 3.16(0.88) |
|  | Number of Peaks of absoulte Value | 3.18(1.01) | 3.14(1.07) | 3.18(0.86) |
| Foot Progression Moment | Maximum Value during Loading Response to Mid-Stance | -15.23(10.74) | -11.37(11.08) | -11.51(8.2) |
|  | Minimum Value during Terminal Stance | -11(10.54) | -7.77(10.82) | -8.16(8.18) |
|  | Maximum Value during Pre-Swing to Initial Swing | -17.58(10.97) | -14.39(10.93) | -14.86(7.73) |
|  | Minimum Value during Terminal Swing | 1(0.59) | 0.99(0.61) | 0.97(0.85) |
|  | Area Under the Curve of Stance Phase | 0.61(0.62) | 0.58(0.79) | 0.51(0.95) |
|  | Area Under the Curve | 1.18(0.69) | 1.16(0.76) | 1.16(1.07) |
|  | Area Under the Curve of Absolute Value During Stance Phase | 0(0.01) | 0(0.01) | 0(0.01) |
|  | Area Under the Curve of Absolute Value | 33.88(24.8) | 33.45(31.23) | 32.97(37.5) |
|  | Root Mean Square | 33.93(24.63) | 33.47(31.08) | 33.07(37.32) |
|  | Mean | 37.26(20.37) | 39.59(23.67) | 40.28(30.37) |
|  | Variance | 38.14(20.58) | 40.35(23.91) | 41.24(30.56) |
|  | Standard Deviation | 0.57(0.3) | 0.59(0.35) | 0.6(0.45) |
|  | Kurtosis | 0.34(0.24) | 0.33(0.31) | 0.33(0.37) |
|  | Peak2RMS | 0.25(0.35) | 0.27(0.31) | 0.32(0.54) |
|  | Maximum - Minimum | 0.44(0.23) | 0.45(0.26) | 0.46(0.33) |
|  | Upper Bound of Autocorrelation | 2.27(0.62) | 2.18(0.6) | 2.42(0.85) |
|  | Lower Bound of Autocorrelation | 2.2(0.23) | 2.17(0.25) | 2.25(0.29) |
|  | Absolute Value of Area Under the Curve During Stance Phase | 1.32(0.68) | 1.34(0.75) | 1.42(0.94) |
|  | Absolute Value of Area Under the Curve | 0.44(0) | 0.44(0) | 0.44(0) |
|  | Mean of Absolute Value | -0.44(0.0013) | -0.44(0.0020) | -0.44(0.0018) |
|  | Variance of Absolute Value | 36.46(20.8) | 38.69(24.4) | 38.42(31.78) |
|  | Standard Deviation of Absolute Value | 36.47(20.67) | 38.65(24.31) | 38.44(31.64) |
|  | Kurtosis of Absolute Value | 0.38(0.2) | 0.4(0.24) | 0.41(0.3) |
|  | Peak2RMS of Absolute Value | 0.23(0.34) | 0.26(0.3) | 0.31(0.52) |
|  | Absolute Maximum - Absolute Minimum | 0.43(0.23) | 0.44(0.26) | 0.44(0.34) |
|  | Time instants of the mid-reference level crossings | 2.32(0.67) | 2.21(0.63) | 2.47(0.92) |
|  | Mid-reference level | 2.2(0.23) | 2.17(0.25) | 2.25(0.29) |
|  | Minimum distance between signals | 1.24(0.67) | 1.27(0.75) | 1.32(0.95) |
|  | upper cumulative sums drifted beyond five standard deviations above a target mean | 1.51(0.58) | 1.46(0.62) | 1.65(0.55) |
|  | lower cumulative sums drifted beyond five standard deviations below a target mean | 0.53(0.42) | 0.48(0.48) | 0.46(0.61) |
|  | index at which the mean of curve changes most significantly | 35.92(20.12) | 38.33(23.25) | 38.74(29.6) |
|  | Area Under the Curve of Power Spectral Density | 24.28(5.95) | 23.41(10.4) | 21.53(11.71) |
|  | Maximum Value of Area Under the Curve of Power Spectral Density | 54.03(25.29) | 53.71(24.62) | 45.45(26.46) |
|  | Occupied bandwidth | 56.94(11.22) | 55.61(13.8) | 53.36(14.45) |
|  | Lower bandwidth frequency bounds | 0.25(0.35) | 0.27(0.31) | 0.32(0.53) |
|  | Upper bandwidth frequency bounds | 19.96(29.05) | 22.28(25.92) | 26.7(46.01) |
|  | Power stored in bandwidth | 1.3(0.31) | 1.38(0.36) | 1.45(0.43) |
|  | Power spectrum | 0(0) | 0(0) | 0(0) |
|  | Max Spectrum Frequencies | 1.31(0.31) | 1.38(0.36) | 1.46(0.43) |
|  | Area Under the Curve of Power Measurment in dB | 20.28(123.94) | 18.99(54.44) | 45.13(192.22) |
|  | Area Under the Curve of Absoulute Value of Power Measurment in dB | 0.03(0.05) | 0.03(0.04) | 0.04(0.07) |
|  | Maximum Value of Power Measurment in dB | 1.53(2.25) | 1.76(2.11) | 2.18(3.79) |
|  | Number of Peaks | -Inf | -Inf | -Inf |
|  | Number of Peaks of absoulte Value | Inf(NaN) | Inf(NaN) | Inf(NaN) |
|  | Minimum Value during Terminal Stance to Pre-Swing | -Inf | -Inf | -Inf |
|  | Maximum Value during Pre-Swing to Initial Swing | 3.72(1.6) | 3.96(1.73) | 3.93(1.41) |
|  | Minimum Value Mid-Swing to Terminal Swing | 5(1.81) | 5.17(1.98) | 5.24(1.66) |
| Spatiotemporal | Total Cadence | 105.92(11.34) | 100.79(13.28) | 105.09(15.22) |
|  | Total Speed | 85.41(18.25) | 75.43(21.82) | 81.87(17.44) |
|  | Total Stride Length | 96.29(15.64) | 89.43(18.79) | 93.53(15.53) |
|  | Total_Step Width | 11.53(2.98) | 11.49(3.24) | 12.15(3.64) |
|  | Right Leg Cadence | 105.81(11.47) | 100.76(13.35) | 105.25(15) |
|  | Right Leg Speed | 85.82(18.49) | 76.31(22.02) | 82.31(17.66) |
|  | Right Leg Stride Length | 96.73(15.76) | 89.74(18.99) | 93.78(15.83) |
|  | Right Leg Step Width | 48.24(8.25) | 45(9.52) | 47.24(7.98) |
|  | Right Leg Single Support | 35.49(2.72) | 33.67(4.38) | 35.27(3.14) |
|  | Duration of single limb support phase | 35.5(2.72) | 33.58(4.38) | 35.34(3.14) |
|  | Timing of initial double limb support | 14.61(3.05) | 16.37(4.38) | 14.42(2.96) |
|  | Right Leg Total Support | 65.27(3.59) | 66.21(4.17) | 64.51(3.66) |
|  | Timing of initial double limb support | 14.61(3.05) | 16.37(4.38) | 14.42(2.96) |
|  | Right Leg Weight Release | 14.9(2.76) | 16.17(3.96) | 14.94(2.97) |
|  | Right Leg Stance | 65.25(3.62) | 66.22(4.18) | 64.47(3.66) |
|  | Right Leg Swing | 34.73(3.59) | 33.79(4.17) | 35.49(3.66) |
