# Supplementary material for: A machine learning-based diagnostic model associated with knee osteoarthritis severity
Source: Sci Rep. 2020 Sep 25;10:15743. doi: 10.1038/s41598-020-72941-4 (PMC7519044; doi:10.1038/s41598-020-72941-4)
Supplement: Supplementary file 2 — Supplementary Table 2. [file 41598_2020_72941_MOESM2_ESM.docx]

A machine learning-based diagnostic model associated with knee osteoarthritis severity

Soon Bin Kwon,^1^ Yunseo Ku,^2^ Hyuk-soo Han^3^, Myung Chul Lee^3^, Hee Chan Kim,^1,4,5^ and Du Hyun Ro^3^

^1^Interdisciplinary Program in Bioengineering, Seoul National University, Seoul, Korea;

^2^Department of Biomedical Engineering, College of Medicine, Chungnam National University, Daejeon, Korea

^3^Department of Orthopedic Surgery, Seoul National University Hospital, Seoul National University College of Medicine;

^4^Institute of Medical & Biological Engineering, Medical Research Center, Seoul National University College of Medicine, Seoul, Korea;

^5^Department of Biomedical Engineering, Seoul National University College of Medicine, Seoul, Korea

**Supplementary Table 2**. Mean and standard deviation of selected features significantly different for severity of physical function groups

| Gait Parameter | Feature | Mild | Moderate | Severe |
| --- | --- | --- | --- | --- |
| Knee Extension Moment | Kurtosis | 2.22(0.52) | 1.99(0.44) | 2.05(0.51) |
|  | Peak2RMS | 2.14(0.3) | 1.99(0.27) | 2(0.29) |
| Knee Flexion Angle | Variance | 276.18(110.38) | 232.79(110.76) | 217.16(115.89) |
|  | Standard Deviation | 16.28(3.3) | 14.71(4.01) | 14(4.6) |
|  | Maximum - Minimum | 51.17(8.92) | 46.77(11.41) | 44.62(12.88) |
|  | Area Under the Curve of Power Spectral Density | 274.11(109.4) | 230.96(109.81) | 215.53(114.97) |
| Hip Rotation Moment | Standard Deviation | 2.67(0.62) | 2.39(0.52) | 2.38(0.57) |
| Hip Flexion Angle | Lower Bound of Autocorrelation | -0.44(0) | -0.43(0) | -0.43(0) |
|  | Bandwidth Frequency Bounds | 0(0) | 0(0) | 0(0) |
| Ankle Plantarflexion Moment | Minimum Value during Loading Response | -0.6(0.54) | -0.35(0.46) | -0.33(0.47) |
|  | Maximum value during initial Swing | -0.31(0.11) | -0.23(0.14) | -0.25(0.1) |
|  | Maximum - Minimum | 11.49(3.58) | 9.75(3.52) | 10.05(2.76) |
| Pelvic Obliquity Angle | Minimum value during Terminal Stance to Pre-Swing | 3.63(0.84) | 3.24(0.88) | 3.24(0.87) |
| Hip Adduction Angle | Area Under the Curve during Stance Phase | -1.6(4.18) | -3.74(4.85) | -5.16(5.4) |
|  | Standard Deviation of Absolute Value | 302.59(273.69) | 414.27(305.87) | 485.84(335.25) |
| Foot Progression Angle | Average of Absolute Value | -0.44(0) | -0.44(0) | -0.44(0) |
| Hip Power | Minimum Value during Mid-Stance | -1.33(1.97) | -0.98(2.01) | -0.2(1.89) |
|  | Maximum Value during Terminal Stance | 4.78(3.89) | 3.13(3.49) | 3.27(3.04) |
|  | Area Under the Curve | 122.14(109.26) | 102.48(103.02) | 158.5(117.82) |
|  | Maximum - Minimum | 11.68(4.85) | 9.27(5.02) | 10.26(4.37) |
|  | Distance between Stance and Swing Phase using Dynamic Time Wrapping | 128.4(61.88) | 95.71(64.42) | 89.72(50.57) |
|  | Maximum Value during Mid-Swing | 6.83(3.16) | 5.16(3.26) | 5.71(2.88) |
| Knee Power | Maximum Value during Terminal Swing | -7.5(3.56) | -5.91(3.48) | -5.93(3.44) |
| Ankle Power | Kurtosis | 6.73(1.15) | 6.08(1.51) | 5.99(1.26) |
|  | Peak2RMS | 3.44(0.32) | 3.27(0.42) | 3.23(0.38) |
|  | Maximum - Minimum | 21.16(8.71) | 16.64(9.05) | 18.21(7.38) |
|  | Lower Bound of Autocorrelation | -0.43(0) | -0.43(0) | -0.43(0) |
|  | Occupied Bandwidth | 0.89(0.26) | 1.08(0.34) | 1.02(0.31) |
|  | Bandwidth frequency bound | 0.89(0.26) | 1.08(0.34) | 1.02(0.31) |
| Knee Varus Angle | Maximum Value during Mid-Stance | 5.27(4.51) | 8.75(6.11) | 9.26(6.09) |
|  | Maximum Value during Terminal Stance | 5.03(4.38) | 8.42(6.09) | 8.81(6.14) |
|  | Area Under the Curve of Stance Phase | 270.17(275.27) | 489.2(379.61) | 508.5(385.48) |
|  | Area Under the Curve | 328.41(420.85) | 639.29(529.35) | 687.18(571.07) |
|  | Root Mean Square (RMS) | 5.15(3.03) | 7.73(3.9) | 8.37(4.14) |
|  | Peak2RMS | 1.73(0.5) | 1.42(0.34) | 1.45(0.38) |
|  | Mid-reference level | 304.65(237.56) | 533.52(296.26) | 558.19(310.67) |
|  | Area Under the Curve of Power Spectrum | 0.93(1.29) | 2.19(2.27) | 2.47(2.4) |
|  | Maximum Value during Terminal Swing | 5.4(4.34) | 7.83(4.98) | 8.28(5.36) |
|  | Minimum Value during Loading Response | 3.06(4.15) | 6.13(5.49) | 6.39(5.61) |
| Spatiotemporal | Total Speed | 85.76(18.04) | 75.42(21.63) | 79.72(19.58) |
|  | Duration of single limb support phase | 35.56(2.68) | 33.61(4.34) | 34.7(3.69) |
|  | Timing of initial double limb support | 14.6(3.06) | 16.37(4.32) | 14.97(3.65) |
