# Supplementary material for: A machine learning-based diagnostic model associated with knee osteoarthritis severity
Source: Sci Rep. 2020 Sep 25;10:15743. doi: 10.1038/s41598-020-72941-4 (PMC7519044; doi:10.1038/s41598-020-72941-4)
Supplement: Supplementary file 3 — Supplementary Table 3. [file 41598_2020_72941_MOESM3_ESM.docx]

A machine learning-based diagnostic model associated with knee osteoarthritis severity

Soon Bin Kwon,^1^ Yunseo Ku,^2^ Hyuk-soo Han^3^, Myung Chul Lee^3^, Hee Chan Kim,^1,4,5^ and Du Hyun Ro^3^

^1^Interdisciplinary Program in Bioengineering, Seoul National University, Seoul, Korea;

^2^Department of Biomedical Engineering, College of Medicine, Chungnam National University, Daejeon, Korea

^3^Department of Orthopedic Surgery, Seoul National University Hospital, Seoul National University College of Medicine;

^4^Institute of Medical & Biological Engineering, Medical Research Center, Seoul National University College of Medicine, Seoul, Korea;

^5^Department of Biomedical Engineering, Seoul National University College of Medicine, Seoul, Korea

**Supplementary Table 3**. Mean and standard deviation of selected features significantly different for severity of pain groups

| Gait Parameter | Features | Mild | Moderate | Severe |
| --- | --- | --- | --- | --- |
| Ankle Plantarflexion Moment | Minimum Value during Loading Response | -0.57(0.57) | -0.33(0.43) | -0.27(0.39) |
| Hip Adduction Angle | Area Under the Curve during Stance Phase | -1.78(4.75) | -4.4(4.64) | -4.53(3.57) |
| Hip Power | Maximum - Minimum | 10.97(4.94) | 9.57(5.04) | 10.34(2.69) |
| Knee Varus Angle | Maximum Value during Mid-Stance | 6(5.67) | 8.87(5.22) | 9.37(5.77) |
|  | Area Under the Curve of Stance Phase | 318.08(355.54) | 489.16(319.75) | 510.58(373.88) |
|  | Area Under the Curve | 393.46(519.85) | 643.7(464.37) | 721.71(544.52) |
|  | Peak2RMS | 1.67(0.48) | 1.45(0.37) | 1.4(0.29) |
|  | Mid-reference level | 361.04(298.89) | 513.39(281.07) | 563.93(291.7) |
|  | Maximum Value during Terminal Swing | 5.77(4.95) | 8.03(4.63) | 8.52(5.18) |
|  | Minimum Value during Loading Response | 3.73(5.21) | 6.13(4.69) | 6.31(5.39) |
