# Supplementary material for: A machine learning-based diagnostic model associated with knee osteoarthritis severity
Source: Sci Rep. 2020 Sep 25;10:15743. doi: 10.1038/s41598-020-72941-4 (PMC7519044; doi:10.1038/s41598-020-72941-4)
Supplement: Supplementary file 4 — Supplementary Table 4. [file 41598_2020_72941_MOESM4_ESM.docx]

A machine learning-based diagnostic model associated with knee osteoarthritis severity

Soon Bin Kwon,^1^ Yunseo Ku,^2^ Hyuk-soo Han^3^, Myung Chul Lee^3^, Hee Chan Kim,^1,4,5^ and Du Hyun Ro^3^

^1^Interdisciplinary Program in Bioengineering, Seoul National University, Seoul, Korea;

^2^Department of Biomedical Engineering, College of Medicine, Chungnam National University, Daejeon, Korea

^3^Department of Orthopedic Surgery, Seoul National University Hospital, Seoul National University College of Medicine;

^4^Institute of Medical & Biological Engineering, Medical Research Center, Seoul National University College of Medicine, Seoul, Korea;

^5^Department of Biomedical Engineering, Seoul National University College of Medicine, Seoul, Korea

**Supplementary Table 4**. Mean and standard deviation of selected features significantly different for severity of stiffness groups

| Gait Parameter | Feature | Mild | Moderate | Severe |
| --- | --- | --- | --- | --- |
| Knee Flexion Angle | Variance | 270.93(115.19) | 234.88(98.35) | 204.49(111.54) |
|  | Standard Deviation | 16.05(3.66) | 14.91(3.58) | 13.59(4.47) |
|  | Maximum - Minimum | 50.36(10.08) | 47.43(10.27) | 43.58(12.6) |
|  | Area Under the Curve of Power Spectral Density | 268.86(114.17) | 233.05(97.54) | 202.93(110.65) |
| Hip Rotation Moment | Standard Deviation | 2.6(0.63) | 2.47(0.5) | 2.3(0.5) |
| Adduction Angle | Area Under the Curve during Stance Phase | -2.28(4.6) | -4.43(4.19) | -4.75(5.7) |
| Knee Varus Angle | Maximum Value during Mid-Stance | 6.24(5.84) | 9.3(4.9) | 9.19(5.8) |
|  | Maximum Value during Terminal Stance | 5.88(5.77) | 9.08(4.69) | 8.83(5.88) |
|  | Area Under the Curve of Stance Phase | 326.5(360.02) | 523.73(300.28) | 513.37(371.02) |
|  | Area Under the Curve | 410.21(523.81) | 684.98(452.15) | 693.52(511.61) |
|  | Root Mean Square (RMS) | 6.12(3.76) | 7.88(3.64) | 8.07(4.1) |
|  | Peak2RMS | 1.63(0.47) | 1.44(0.37) | 1.41(0.34) |
|  | Mid-reference level | 386.13(294.82) | 538.34(272.69) | 551.15(311.33) |
|  | Area Under the Curve of Power Spectrum | 1.43(1.89) | 2.15(1.85) | 2.41(2.72) |
|  | Maximum Value during Terminal Swing | 6.01(4.95) | 8.12(4.34) | 8.42(5) |
|  | Minimum Value during Loading Response | 3.88(5.37) | 6.46(4.27) | 6.62(5.18) |
